# Ligands reshape the compactness, stability, and topology of telomeric G-quadruplex dimers

**DOI:** 10.1093/nar/gkag403

**Published:** 2026-05-04

**Authors:** Luca Bertini, Valeria Libera, Valentina Arciuolo, Mattia Trapella, Simona Marzano, Deniz Mostarac, Giorgio Schirò, Caterina Petrillo, Concetta Giancola, Cristiano De Michele, Jussara Amato, Lucia Comez, Bruno Pagano, Alessandro Paciaroni

**Affiliations:** Dipartimento di Fisica e Geologia, Università degli Studi di Perugia, Via Alessandro Pascoli, Perugia 06123, Italy; Dipartimento di Fisica e Geologia, Università degli Studi di Perugia, Via Alessandro Pascoli, Perugia 06123, Italy; Department of Pharmacy, University of Naples Federico II, Naples 80131, Italy; Dipartimento di Fisica e Geologia, Università degli Studi di Perugia, Via Alessandro Pascoli, Perugia 06123, Italy; Department of Pharmacy, University of Naples Federico II, Naples 80131, Italy; Dipartimento di Fisica, Università di Roma La Sapienza, Piazzale Aldo Moro 2, Roma 00185, Italy; CNRS, Institut de Biologie Structurale, 71 Avenue des Martyrs, Grenoble 38044, France; Dipartimento di Fisica e Geologia, Università degli Studi di Perugia, Via Alessandro Pascoli, Perugia 06123, Italy; Department of Pharmacy, University of Naples Federico II, Naples 80131, Italy; Dipartimento di Fisica, Università di Roma La Sapienza, Piazzale Aldo Moro 2, Roma 00185, Italy; Department of Pharmacy, University of Naples Federico II, Naples 80131, Italy; CNR-Istituto Officina dei Materiali (IOM), Unità Perugia, Perugia 06123, Italy; Department of Pharmacy, University of Naples Federico II, Naples 80131, Italy; Dipartimento di Fisica e Geologia, Università degli Studi di Perugia, Via Alessandro Pascoli, Perugia 06123, Italy

## Abstract

G-quadruplexes (G4s) are noncanonical nucleic acid structures especially abundant in telomeres, where they can assemble into higher-order multimers. Stabilization of these assemblies is recognized as a promising strategy for suppressing tumor proliferation. Elucidating their structural stability and topological responses to small-molecule binding is therefore essential for advancing their therapeutic potential. Here, we employ a multi-technique approach, combining circular dichroism and small-angle X-ray scattering with extremely coarse-grained simulations, to characterize the impact of four well-established ligands on telomeric G4 dimers. All ligands promote significant stacking interactions between the G4 units in the dimer, leading to more compact complexes, with the fraction of stacked units exhibiting a linear dependence on the effective distance between their centers of mass. Nonetheless, each ligand predominantly interacts with a single G4 unit at a time, inducing thermal stabilization of the monomers within the dimer comparable to that of the corresponding monomeric species. Strikingly, the extent of ligand-induced topological rearrangements observed in the complexes is associated with their stability, but not with compactness. These results provide new insights into ligand interactions with G4 dimers and offer mechanistic guidance for the design of G4 multimer stabilizers.

## Graphical Abstract

**Graphical Abstract:**
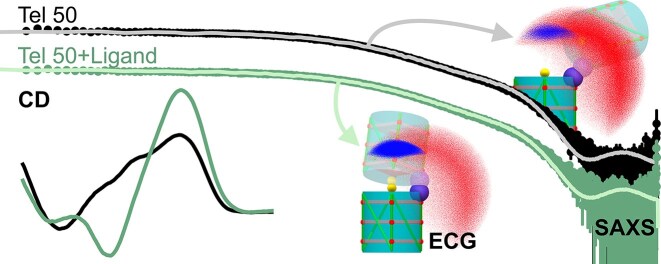

## Introduction

**Figure 1.**
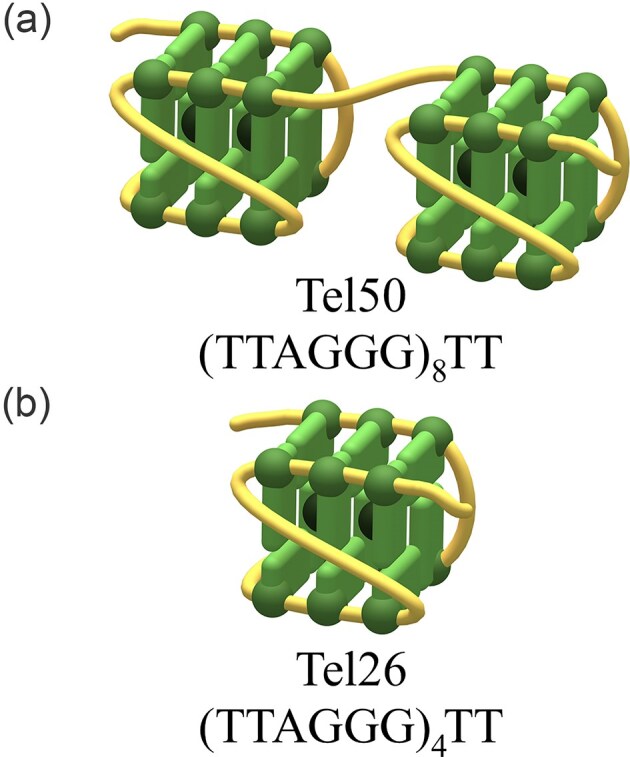
Schematic representation of the (**a**) dimeric and (**b**) monomeric G4 structures formed by the human telomeric sequences Tel50 and Tel26, respectively.

The human telomere is a region located at the ends of linear chromosomes, composed of tandem repeats of the sequence d(TTAGGG)_n_ [1]. Its length typically ranges from 5 to 15 kilobases in somatic cells and includes an extended single-stranded 3′ G-rich overhang of few hundred bases [2]. Telomeres protect the ends of chromosomal DNA from gradual degradation and help maintain the structural integrity of linear chromosomes [3]. However, with each cell division, telomeres usually progressively shorten as part of the aging process, until they become too short to fulfill their protective role [4]. By triggering cellular senescence and limiting cell proliferation, telomere shortening has evolved as a major tumor suppression mechanism [5]. In cancer cells, telomere length is maintained through reactivation of the human telomerase reverse transcriptase (hTERT) [6], allowing them to bypass replicative senescence and divide indefinitely. G-quadruplexes (G4s), four-stranded nucleic acid structures formed by stacked G-tetrads in guanine-rich sequences, can modulate telomere-extending activity when they form in the telomeric overhang [7]. Stabilized G4s can perturb telomere structure and function, inducing replication stress, activating DNA damage responses, and promoting chromosomal instability, ultimately triggering senescence or apoptosis in cancer cells [8]. Because of these findings, G4s have attracted considerable attention as potential anti-cancer targets in drug design [9]. G4s formed by human telomeric sequences have often been studied in their monomeric state [10–14], despite clear evidence that the extended single-stranded 3′ G-rich overhang can give rise to G4 multimers, also referred to as G4 higher-order structures [15]. The structural characterization of G4s is inherently challenging even in their monomeric form due to pronounced polymorphism, which is strongly influenced by factors such as oligonucleotide sequence, specific nature and concentration of cations, molecular crowding agents, and DNA concentration [16, 17]. Studying telomeric G4 multimers is even more difficult, as additional complexity arises from potential interactions between individual G4 units [15]. Identifying additional binding sites and potential cooperative effects that govern small molecule interactions with G4 multimers is a crucial step toward designing stronger and more stable complexes, which are desirable traits for effective drug candidates. In addition, ligands specifically recognizing the unique inter-unit topologies and interfaces of multimers could exhibit higher selectivity for telomeric targets over other G4-forming sequences elsewhere in the genome. While previous studies have investigated the ability of both monomeric [18–22] and dimeric ligands [23–28] to selectively interact with G4 dimers, the effects of ligands on higher-order architectures and their stability remain poorly understood due to the multiple factors involved. In this study, we investigated the effect of G4 binders on the compactness, topology, and stability of a 50-nucleotide-long G-rich telomeric sequence (Tel50), capable of forming a dimer composed of two covalently linked G4 units (Fig. 1a). This system potentially mimics biologically relevant telomeric DNA and may occur within chromosomes. Ligand-binding behavior was analyzed in comparison with the corresponding single G4-forming sequence (Tel26, Fig. 1b) using a multi-technique approach that combines circular dichroism (CD) and small-angle X-ray scattering (SAXS) with extremely coarse-grained Monte Carlo (ECG-MC) simulations.

To this end, we selected a set of well-known G4 ligands: BRACO-19, PhenDC3, RHPS4, and SYUIQ-5 (Supplementary Fig. S1), which have distinct structural features and binding modes [29–32]. Our results show that all four ligands, to varying degrees, promote significant stacking between G4 units within the dimer, resulting in more compact complexes. Importantly, the extent of ligand-induced topological rearrangements within the G4 units tracks with the increase in thermal stability, but not with the degree of complex compactness.

## Materials and methods

### Materials

CPG supports DNA phosphoramidites, and all reagents for oligonucleotide synthesis were purchased from Link Technologies (Bellshill, UK) and used without further purification. All buffers were prepared using Milli-Q ultrapure water and filtered before using. BRACO-19, PhenDC3, RHPS4, and SYUIQ-5 (Supplementary Fig. S1), as well as all other chemicals, were purchased from Merck KGaA (Darmstadt, Germany).

### Oligonucleotide synthesis and sample preparation

The DNA sequences d(TTA GGG TTA GGG TTA GGG TTA GGG TT) (Tel26) and d(TTA GGG TTA GGG TTA GGG TTA GGG TTA GGG TTA GGG TTA GGG TTA GGG TT) (Tel50) were chemically synthesized at a 1 μmol scale in DMT-ON mode using an HT-16 DNA/RNA synthesizer (K&A Laborgeraete) and standard β-cyanoethylphosphoramidite solid-phase chemistry [33, 34]. Following synthesis, the oligonucleotides were cleaved from the support and deprotected in concentrated aqueous ammonia at 55°C for 17 h. Purification was performed using Glen-Pak cartridges following the manufacturer’s protocol. The purified samples were dried by vacuum centrifugation, resuspended in 200 mM LiCl, and desalted using Amicon-15 centrifuge filters (3.0 kDa MWCO). The oligonucleotides were then lyophilized and resuspended in 5 mM KH_2_PO_4_/K_2_HPO_4_ buffer at pH 7.0, supplemented with 20 mM KCl to prepare stock solutions for CD, UV, and SAXS experiments. DNA concentrations were determined at 90°C by UV absorbance at 260 nm using a Jasco V-730 spectrophotometer (JASCO Inc., Tokyo, Japan) equipped with ETCS-761 Peltier cell holder, and molar extinction coefficients were calculated as previously reported [35]. Finally, samples were annealed by heating to 90°C for 5 min, followed by slow cooling to room temperature overnight and storage at 4°C for 24 h before use. To minimize aggregate contributions, the annealed Tel50 sample was purified by size-exclusion chromatography before experiments. Ligand complexes with Tel26 were examined at G4-to-ligand stoichiometric ratios of 1:1 and 1:2, while complexes with Tel50 were analyzed at ratios of 1:2, 1:3, and 1:4.

### Size-exclusion chromatography

To isolate Tel50 and remove aggregates, the annealed DNA solution (0.5 mM) in 5 mM KH_2_PO_4_/K_2_HPO_4_ buffer at pH 7.0, supplemented with 20 mM KCl, was purified by size-exclusion chromatography (SEC). Purification was performed on a Superdex 75 16/600 column equilibrated with the same buffer and operated at a flow rate of 0.5 ml/min using an ÄKTA pure system. A 500 μl aliquot of the 0.5 mM Tel50 DNA sample was injected for separation, and elution was monitored by UV absorbance at 260 nm. Fractions of 0.5 ml with a 100–150 μM DNA concentration were collected. All samples used for subsequent experiments were prepared from these SEC-purified stock solutions by dilution to the concentrations required for each technique. Thus, all measurements were performed on identically prepared material, ensuring consistency and reproducibility across all experiments.

### Polyacrylamide gel electrophoresis

Native polyacrylamide gel electrophoresis (PAGE) (12.5%) gels were prepared using a 29:1 acrylamide/bisacrylamide solution in a 1× TBE buffer (Tris–Borate–EDTA), pH 7.0. Electrophoresis was carried out at 4°C and 120 V for 1 h. Oligonucleotides were diluted to 5 μM in a 5 mM KH_2_PO_4_/K_2_HPO_4_ buffer at pH 7.0, supplemented with 20 mM KCl, and analyzed to confirm the purity and molecularity of the investigated G4 structures. A 10% (final) glycerol/TBE solution was added to facilitate sample loading into the wells. Bands were visualized by using GelRed^®^ nucleic acid stain. Native PAGE analysis was repeated over time to assess the stability of the SEC-purified samples. The telomeric sequence mTel24, previously demonstrated to form a monomolecular hybrid G4 structure in solution (PDB: 2GKU), was included as a structural control.

### Small-angle X-ray scattering

Experiments. SAXS experiments were performed at the BM29 beamline, the dedicated BioSAXS infrastructure for the characterization of biological macromolecules in solution at the European Synchrotron Radiation Facility (ESRF) in Grenoble, France (https://www.esrf.fr/BM29-BioSAXS).

The instrument is equipped with a Pilatus3 2M detector operating in vacuum and a double multilayer monochromator with an energy bandpass of ~10^−2^. Tel26 and Tel50 were used at final concentrations of 90 µM and 45 µM, respectively. SAXS profiles were acquired at a temperature of 20°C using an incident beam with an energy (E) of 12.5 keV, corresponding to an incident wavelength (λ) of 0.99 Å. The investigated wave-vector transfer range spanned 0.044–5.21 nm^−1^.

The SAXS curves of the corresponding buffers and ligands alone, at all investigated stoichiometric ratios, were also collected to enable accurate subtraction of background contributions. To improve statistical accuracy, each curve was obtained by averaging over 10 frames for samples and ligands alone, and over 20 frames for the buffer. The acquisition time for each frame was 1 s.

Modeling of SAXS data. SAXS experiments consist of measuring the scattered intensity I(*Q*), which is proportional to differential scattering cross-section *dΣ/dΩ*(*Q*). Here, *Q* is the wavevector transfer between the incident beam and the sample. For *N* biomolecules in solution, where the orientation of the scattering particles is random, the scattering cross section can be written as [36]:


(1) \begin{eqnarray*}
\frac{{d\sigma }}{{d\Omega }}\left( Q \right) = \frac{N}{V}\ \Delta {{\rho }^2}\ V_P^2\ P\left( Q \right)S\left( Q \right),
\end{eqnarray*}

where *Δρ* is the scattering length density contrast between the particle and the solvent, *V_P_* is the particle volume, and *N* is the number of particles. *P*(*Q*) is the form factor depending on the shape and dimension of the scattering particle, while *S*(Q) is the inter-particle structure factor. In the absence of attractive/repulsive interactions between particles and non-specific aggregation, in the low-Q region one can apply the Guinier approximation to estimate the gyration radius *R*_g_ of the biomolecules, i.e. the root-mean square of the distance of all electrons from the center of gravity of the system [37]:


\begin{eqnarray*}
\frac{{d\sigma }}{{d\Omega }}( Q )\sim{\mathrm{exp}}( { - \frac{{{{Q}^2}R_g^2}}{3}} ).
\end{eqnarray*}

The *S*(*Q*) is given by the ensemble average:


(2) \begin{eqnarray*}
S\left( Q \right) = \frac{1}{N}\left\langle {\mathop \sum \limits_{i = 1}^N \mathop \sum \limits_{j = 1}^N \exp \left( {iQ \cdot {{R}_{ij}}} \right)} \right\rangle ,
\end{eqnarray*}

where *R_ij_* is the distance between the centers of mass of particles i and j, while <…> denotes the ensemble average. We remark that Equation (1) is an approximation that is valid under the assumption of spherically symmetric, monodisperse particles with a uniform scattering length density [38].

To provide a quantitative estimate of the ligand-induced compaction of the dimers, we computed the ratio *R*(*Q*) between the SAXS intensities of Tel50, *I*_T_(*Q*), and Tel50-ligand complexes, *I*_T+L_(*Q*). Using Equation (1), we obtain:


(3) \begin{eqnarray*}
R\left( Q \right)\ = \frac{{{{I}_T}\left( Q \right)}}{{{{I}_{T + L}}\left( Q \right)}}\ = \ k\ \frac{{{{P}_T}\left( Q \right)\ {{S}_T}\left( Q \right)}}{{{{P}_{T + L}}\left( Q \right)\ {{S}_{T + L}}\left( Q \right)}},
\end{eqnarray*}

where *k* is a pre-factor that ultimately depends on the ratio between the volumes of the scattering particles. In principle, the monomeric units of Tel50 undergo conformational changes in the presence of ligands, so that we may expect that the two form factors *P_T_*(*Q*) and *P_T+L_*(*Q)* are different. Nevertheless, as mentioned before, the wave-vector transfer range investigated allows only a low-resolution reconstruction of the overall shape of the G4 structure and the surrounding water molecules and cations. Consequently, minor shifts in topology and shape are not expected to cause significant changes in the form factors. Therefore, we assume that *P*_T_(*Q*) = *P_T+L_*(*Q*). As for the structure factor *S*(Q), the ensemble average in Equation (2) includes both averaging over the random orientations of the particles and the distribution of *R*_ij_. Such distribution is unknown *a priori* and its inclusion in Equation (3) might not necessarily result in a closed-form expression. Consequently, we introduce an “effective” ensemble with fixed distance *R* between the monomers’ centers of mass, so that in the case of randomly oriented, non-interacting dimers, we obtain:


(4) \begin{eqnarray*}
S\left( Q \right)\ = \ 2\ \left( {1\ + \ \frac{{sin\left( {QR} \right)}}{{QR}}} \right).
\end{eqnarray*}

Under all these assumptions, we can rewrite Equation (3) as:


(5) \begin{eqnarray*}
R\left( Q \right)\ = k\frac{{1\ + \ sin\left( {Q{{R}_{cm\ T}}} \right)/\left( {Q{{R}_{cm\ T}}} \right)}}{{1\ + \ sin\left( {Q{{R}_{cm\ T + L}}} \right)/\left( {Q{{R}_{cm\ T + L}}} \right)}}.
\end{eqnarray*}

Using Equation (5), we performed a simultaneous fit to the whole dataset, with *R*_cm T_, *R*_cm T+L_, and *k* as fitting parameters, while *R*_T_ was common to the whole dataset.

Determination of the Radius of Gyration. For all samples, *R*_g_ was determined directly from the SAXS data using the AUTORG routine developed by the Svergun group [39]. This procedure is based on statistical criteria for identifying the optimal fitting range to apply the Guinier approximation. In addition, to compute *R*_g_ also in the real space, the *p(r)* distributions were reconstructed by means of indirect Fourier transform methods implemented in the GNOM package [40]. All results are summarized in Supplementary Tables S1 and S2, and representative best-fitting curves for Tel26 and Tel50 are shown in Supplementary Figs S2 and S3. The discrepancy between reciprocal-space and real-space *R*_g_ values for the Tel26 samples ranges from 1% to 3%, consistent with the expected few-percent variation for ideal monodisperse systems, thus confirming the quality of the data. The Tel50 samples show a slightly larger but still acceptable discrepancy (3%–5%), reflecting their moderately increased conformational flexibility as dimers [41].

### Extremely coarse-grained simulations

**Figure 2.**
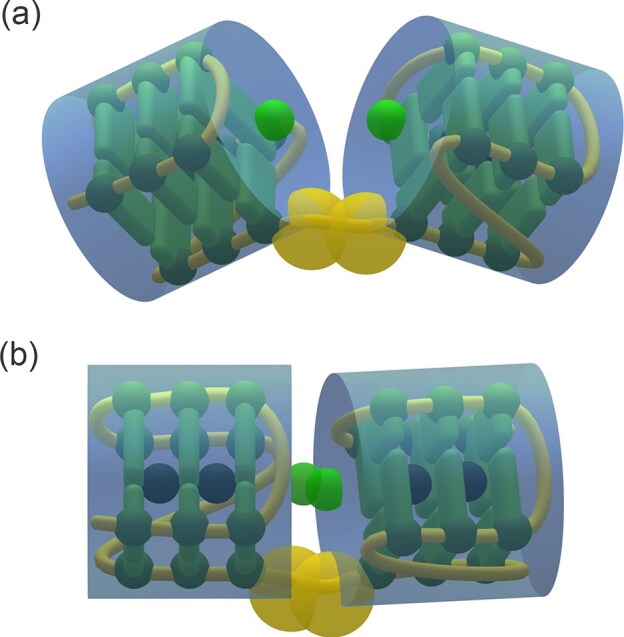
Simulation model using HCs for a G4 dimer in the unstacked (**a**) and stacked (**b**) conformations. The green patches represent stacking interactions, while the yellow patches represent covalent interactions mimicking the TTA linkers.

To investigate how ligands modulate the interactions between G4 units, we employed ECG-MC simulations, adapting a previously established model used to study long telomeric sequences [42, 43]. In this model, each Tel50 dimer is represented by two hard cylinders (HCs) held together via spherical patches of radius *R*_0_ = 0.53 nm, positioned at the edges of the HC bases (see yellow spheres in Fig. 2). The centers of these patches interact through an infinite square-well potential of width 2*R_0_* to mimic the role of TTA linkers in telomeric DNA. Additionally, stacking interactions between two HCs forming a dimer are incorporated through two spherical patches of radius *R*_1_ = 0.265 nm, centered on the HC bases (see green spheres in Fig. 2). The centers of these two patches interact through a finite square-well potential of width 2*R*_1_. The dept u_0_ of this potential well, which governs the stacking interaction strength, is controlled by the dimensionless effective temperature ${{{\boldsymbol{T}}}^*} = {{{\boldsymbol{k}}}_{\boldsymbol{B}}}{\boldsymbol{T}}/{{{\boldsymbol{u}}}_0}$. The model does not account for fine details of G4 [42].

Monte Carlo simulations were performed in the canonical ${\boldsymbol{NV}}{{{\boldsymbol{T}}}^*}$ ensemble using a high-performance algorithm for detecting overlaps between HCs [44, 45]. We simulated a box with periodic boundary conditions at low particle density containing ${\boldsymbol{N}} = 3000$ dimers. Initially, dimers in the stacked position were randomly placed. All simulations were thermalized for at least $5 \times {{10}^5}$ Monte Carlo steps to ensure the total stacking energy reached equilibrium. To compute the simulated SAXS intensity, $50$ independent snapshots were selected from each simulation, corresponding to a total of 50 × 3000 independent dimeric configurations. We verified that increasing the number of configurations does not significantly reduce the uncertainty in the simulated I(Q) compared to the experimental data. In each snapshot, every cylinder was replaced by 100 scattering points randomly distributed within the HC volume [46], ensuring that the simulated SAXS intensity properly accounted for the cylinders’ form factor.

To determine the optimal cylinder dimensions that best reproduce the experimental SAXS intensity, we systematically explored various combinations of cylindrical radii (*R_HC_*) and heights (*H*). *R_HC_* was varied between 1.18 and 1.26 nm in increments of 0.01 nm. For each *R_HC_*, three different *H* were examined, adjusted in 0.06 nm increments around the value that maintains a constant *R*_g_. The best agreement with experimental data was obtained for *R_HC_* equal to 1.26 nm and *H* to 2.64 nm, dimensions that remained consistent across all ligand concentrations. For each combination of *R_HC_* and H, simulations were performed at T* = 0, corresponding to fully stacked dimers, and at T* = ∞, where stacking interactions are absent and dimers stack only randomly. The corresponding SAXS intensities, *I₀(Q)* and *I_∞_(Q)*, were computed and used to fit the experimental profiles via a linear combination:


\begin{eqnarray*}
{\boldsymbol{I}}\left( {\boldsymbol{Q}} \right) = {\boldsymbol{A}}\left( {{{{\boldsymbol{p}}}_{{\boldsymbol{st}}}}{{{\boldsymbol{I}}}_0}\left( {\boldsymbol{Q}} \right) + \left( {1 - {{{\boldsymbol{p}}}_{{\boldsymbol{st}}}}} \right){{{\boldsymbol{I}}}_\infty }\left( {\boldsymbol{Q}} \right)} \right) + {\boldsymbol{bckg}},
\end{eqnarray*}

where *p_st_* is the fraction of dimers in the stacked configuration, *A* is a constant scale factor, and ${\boldsymbol{bckg}}$ a constant background.

### Circular dichroism

CD measurements were carried out on a Jasco J-810 spectropolarimeter and using a 1 mm path-length quartz cuvette. The G4-forming oligonucleotides Tel26 and Tel50 were used at final concentrations of 30 µM and 15 µM, respectively. Spectra were collected over the 220–330 nm wavelength range using a scan speed of 50 nm/min with an 8 s response time and 2 nm bandwidth response (i.e. the range of wavelengths passing through the monochromator slit during measurement). The temperature was gradually increased from 24 to 100°C in 2°C increments using a thermal bath with a rate of 0.5°C/min.

CD spectra of Tel26 and Tel50 in the absence and presence of ligands were quantitatively compared by calculating the root of the sum of squared differences (RSQ) between paired spectra. All spectra were sampled over the same wavelength range and represented as intensity vectors for CD_ligand_ and CD_Tel_.

The spectral dissimilarity was computed as:


\begin{eqnarray*}
{\boldsymbol{RSQ}} = \ \sqrt {\mathop \sum \limits_{\boldsymbol{i}} \ {{{[{\boldsymbol{C}}{{{\boldsymbol{D}}}_{{\boldsymbol{ligand}}}}\left( {{{{\boldsymbol{\lambda }}}_{\boldsymbol{i}}}} \right) - {\boldsymbol{C}}{{{\boldsymbol{D}}}_{{\boldsymbol{Tel}}}}\left( {{{{\boldsymbol{\lambda }}}_{\boldsymbol{i}}}} \right)]}}^2}} ,
\end{eqnarray*}

where the sum runs over all measured wavelengths. The resulting value provides a global quantitative measure of the difference between the CD profiles, reflecting the extent of secondary structure changes induced by ligand binding. The analysis was carried out with a custom in-house script in Python 3.12.10.

### Thermal difference spectra

Thermal difference spectra (TDS) were obtained by recording the UV absorbance spectra in the wavelength range of 220–320 nm at 20 and 90°C and subsequently taking the difference between the two spectra. Spectra were recorded on a Jasco V-730 UV-visible spectrophotometer (JASCO Inc., Tokyo, Japan) equipped with an ETCS-761 Peltier cell holder using quartz cuvette of 0.1 cm path length. Tel26 and Tel50 samples were diluted at concentrations of 30 µM and 15 µM, respectively, using the 5 mM KH_2_PO_4_/K_2_HPO_4_ buffer at pH 7.0 supplemented with 20 mM KCl.

### Nuclear magnetic resonance

1D ^1^H nuclear magnetic resonance (NMR) spectra were recorded on a Bruker Advance NEO NMR spectrometer (Bruker BioSpin, Rheinstetten, Germany), operating at 600 MHz and equipped with a 5 mm QCI cryo-probe set and a cooled SampleJet autosampler. Samples were prepared at 25 µM DNA concentration in 5 mM KH_2_PO_4_/K_2_HPO_4_ buffer at pH 7.0, supplemented with 20 mM KCl, and transferred to 5 mm NMR tubes. D₂O (10%) was added prior to acquisition. NMR spectra were recorded at 25°C using excitation sculpting with pulsed field gradients for water suppression. Each spectrum was acquired with 512 scans and a recovery delay of 1.5 s. Spectra were Fourier transformed, phase adjusted, baseline corrected, and calibrated with respect to the 4,4-dimethyl-4-silapentane-1-sulfonic acid (DSS), used as a reference, assuming a resonance at 0.0 ppm. NMR spectra were processed and analyzed using TopSpin 4.5.0 (Bruker), and MestReNova software.

### Singular value decomposition analysis

Singular value decomposition (SVD) is a technique that factorizes a matrix, D, into three matrices: U, S, and V, such that D = U S V^T^, where V^T^ denotes the transpose of V. In this case, the D matrix contains the CD experimental spectra at different temperatures as its columns. The matrix U consists of basis spectra that, when combined, reconstruct the entire dataset. S is a diagonal matrix, whose diagonal elements, the singular values, indicate the relative contribution of each component. The matrix V contains amplitude vectors as a function of temperature. The procedure for determining the minimum number of spectral components required to accurately reproduce the dataset has been described previously [47]. Essentially, the magnitudes and relative variances of the singular values, along with the autocorrelation coefficients of the U and V matrix vectors, were evaluated against predefined acceptance/rejection criteria. Using an autocorrelation coefficient threshold of 0.8 to indicate statistical significance, three to four significant V vectors were identified and linked to a folded-intermediates-unfolded melting pathway. We found that a three-state unfolding pathway provides a good approximation for all datasets. Therefore, although a four-state model could statistically describe some samples more accurately, the three-state model enables direct and consistent comparison across all samples. The V vectors were then globally fitted to analytical models suitable for investigating the thermodynamics of thermal unfolding. SVD analysis was performed in Python 3.12.10. Matrix factorization was carried out using the NumPy library, whereas the criteria for determining the number of states and the global fitting procedure were implemented in custom in-house scripts.

## Results

### Ligand binding promotes stacked conformations of telomeric dimers

**Figure 3.**
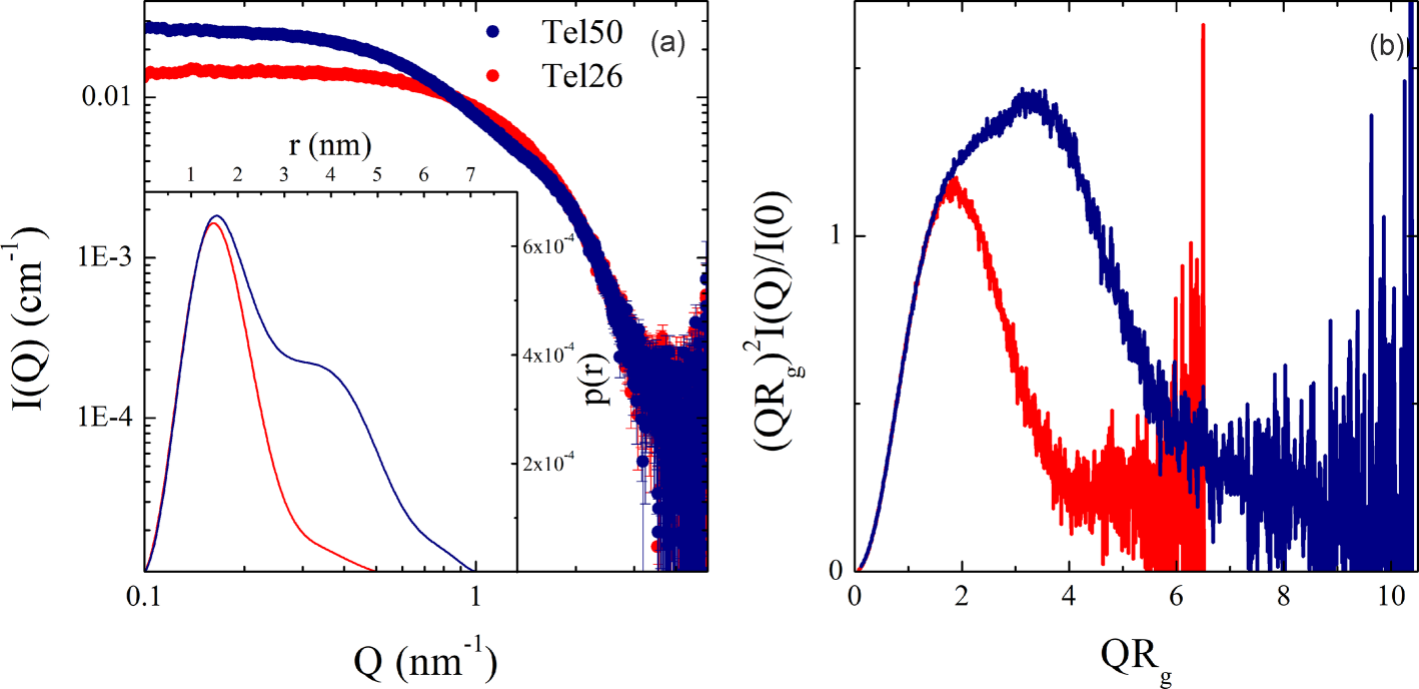
**a**) Comparison between the SAXS signals of Tel26 (red) and Tel50 (blue); the corresponding *R*_g_ estimated by the Guinier approximation are 1.31 ± 0.03 nm and 2.10 ± 0.02 nm, respectively. Inset: pair-distance distributions *p(r)* of Tel26 (red) and Tel50 (blue). (**b**) Dimensionless Kratky plots of Tel26 (red) and Tel50 (blue).

As shown in Fig. 3a, the SAXS intensity profiles of Tel26 and Tel50 exhibit a plateau in the low-Q region, indicative of absent or negligible inter-particle interactions. PAGE analysis of the Tel50 sample before and after SEC purification (Supplementary Fig. S4) further corroborates this observation. SEC-purified samples remain unchanged even after long-term storage (Supplementary Fig. S5), indicating that both the monomeric and dimeric forms are stable over time under the experimental conditions employed. The main structural properties of the investigated samples can be better appreciated in two standard data transformations: the pair-distance distribution p(r) (inset of Fig. 3a) and the Kratky plot (Fig. 3b) [41, 48, 49]. In both representations, the monomeric state of Tel26 G4 can be inferred from a unimodal, bell-shaped curve. By contrast, Tel50 displays a second component consistent with a dimeric structure, confirming the presence of two stable G4 domains. Additionally, the return of the Kratky plots to baseline further supports that both systems adopt a well-folded conformation.

From the SAXS intensity profile of Tel26, a quantitative assessment of the dimensions of the monomeric G4 units can be performed. Given that interparticle interactions can be neglected, the SAXS intensity is directly proportional to the monomer form factor *P*(*Q*). Several closed-form expressions for *P*(*Q*) corresponding to different basic geometries have been reported previously [37]. In earlier work, we demonstrated that a hard cylinder geometry provides an accurate description of the SAXS intensity profiles of G4 monomers while minimizing the number of fitting parameters [42, 43]. Using the same approach, we confirmed that the HC model accurately reproduces the experimental data for Tel26 (Supplementary Fig. S6). Interestingly, we notice that the SAXS profile of the monomeric sequence remained essentially unchanged upon interaction with the investigated ligands (Supplementary Fig. S7), as indicated by the comparable *R*_g_ values (Supplementary Table S1). 

Addressing dimeric structures such as that formed by the Tel50 sequence presents a considerable challenge. Recent studies have shown that long telomeric sequences forming multiple G4s exhibit a certain degree of conformational flexibility, primarily due to the linker regions connecting consecutive units [49, 50]. Consequently, the measured SAXS signal reflects an ensemble of G4 dimers adopting both unstacked and stacked conformations, which are represented in Fig. 2a and b, respectively.

To overcome the lack of analytical expressions describing the SAXS intensity of such systems, we employed an ECG-MC simulation algorithm, recently applied to describe multimeric G4 systems [42]. In this SAXS-guided ECG-MC framework, dimers were modeled as an ensemble of two G4 domains (Fig. 2), each approximated as HCs capable of establishing stacking interactions, as described in the *Extremely Coarse-Grained simulations* section. Remarkably, the experimental SAXS profiles of the investigated samples were accurately reproduced from these ensembles.

**Figure 4.**
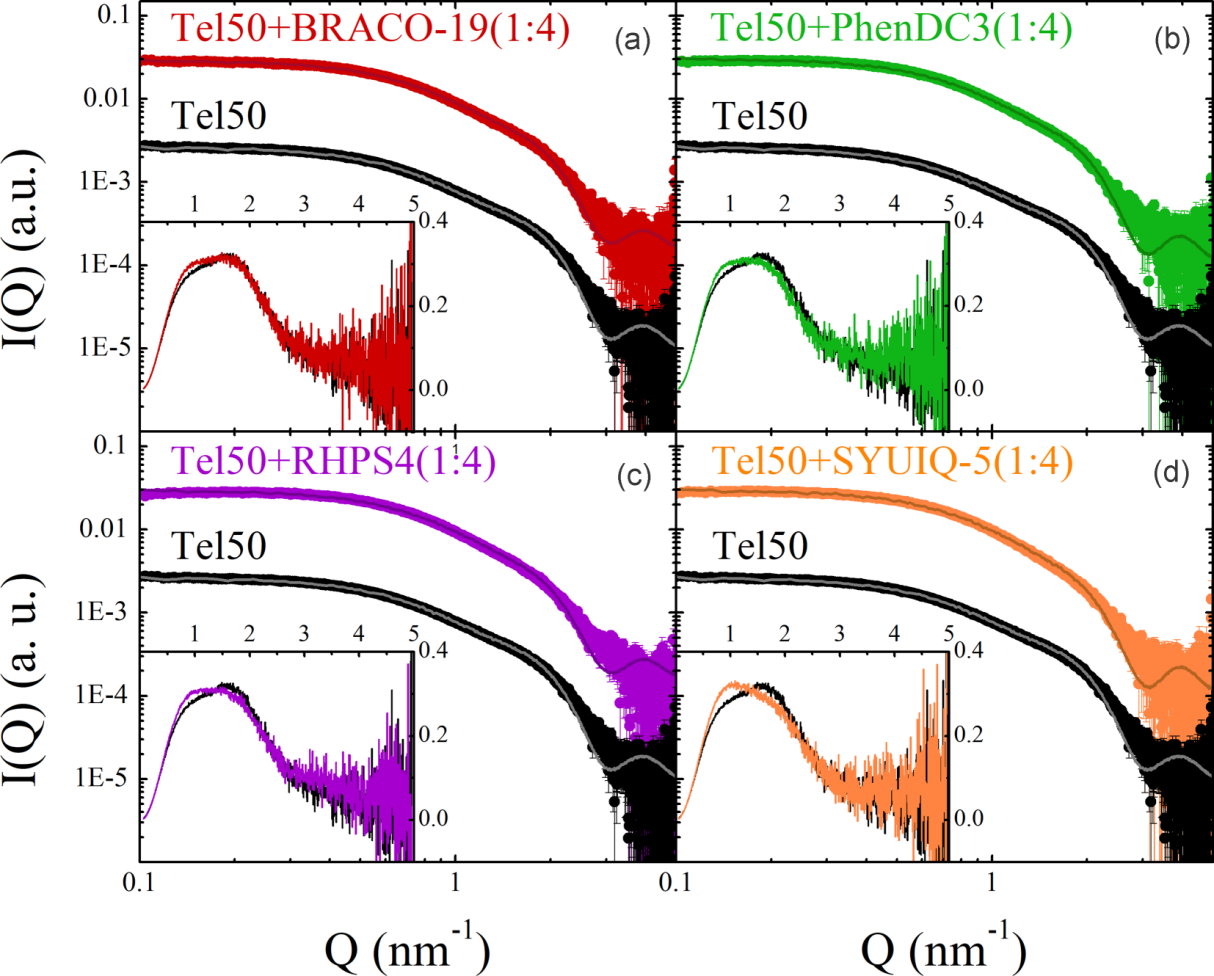
Comparison between the SAXS signals of Tel50 alone and in the presence of (**a**) BRACO-19, (**b**) PhenDC3, (**c**) RHPS4, and (**d**) SYUIQ-5 (1:4 DNA/ligand ratio). The best fitting ECG-MC simulated SAXS profiles are also shown (solid lines). Insets show the corresponding Kratky plots (Q^2^I(Q)/I(0) versus Q) highlighting the structural differences among the samples.

Figure 4 shows the comparison between simulated and experimental SAXS profiles of Tel50 in the absence and presence of ligands at a 1:4 stoichiometric ratio, while the results for 1:2 and 1:3 ratios are reported in Supplementary Fig. S8. The overlap between experimental and simulated curves is excellent in the intermediate to low-Q region, which contains information on the large-scale structural properties, as well as the interaction between the G4 domains. At high-Q, the agreement between the model and the SAXS data is less accurate, though still within experimental uncertainty, reflecting spatial correlations at shorter length scales. This minor discrepancy likely arises from the HC approximation, which, as expected, does not fully capture the exact shape of the G4 unit and its surrounding hydration shell. Best-fit parameters obtained by simulation of experimental SAXS profiles gave a radius *R_HC_* = 1.26 nm and height *H* = 2.64 nm for the HC units in all samples. As observed for the Tel26 monomer, structural differences cannot be discerned due to the potential coexistence of distinct G4 topologies within the dimers. Since all differences in the SAXS curves are attributed primarily to the strength of stacking interactions, we calculated the fraction of stacked conformations *p_st_*, from each best-fitting simulation (Table 1) to enable a quantitative comparison. Notably, *p_st_* is markedly higher in Tel50-ligand complexes than in unbound Tel50 and increases significantly with ligand concentration, thereby indicating ligand-induced promotion of G4–G4 stacking.

**Table 1.** Values of *p*_st_ retrieved from the best-fitting ECG-MC simulations, effective distance between the monomers’ centers of mass *R*_cm_ from Equation (5), and radii of gyration *R*_g_ obtained from the SAXS intensities of the investigated samples

Sample	Ratio	*p*st	*R*cm (nm)	*R*g (nm)
Tel50	-	0.13 ± 0.02	3.59 ± 0.01	2.10 ± 0.02
+BRACO-19	1:2	0.37 ± 0.02	3.50 ± 0.01	2.02 ± 0.02
+BRACO-19	1:3	0.47 ± 0.02	3.44 ± 0.01	2.00 ± 0.03
+BRACO-19	1:4	0.54 ± 0.02	3.42 ± 0.01	1.98 ± 0.02
+ PhenDC3	1:2	0.38 ± 0.02	3.48 ± 0.01	2.03 ± 0.01
+ PhenDC3	1:3	0.56 ± 0.02	3.40 ± 0.01	1.99 ± 0.03
+ PhenDC3	1:4	0.75 ± 0.03	3.33 ± 0.01	1.97 ± 0.02
+ RHPS4	1:2	0.37 ± 0.02	3.50 ± 0.01	2.03 ± 0.01
+ RHPS4	1:3	0.69 ± 0.02	3.34 ± 0.01	2.01 ± 0.02
+ RHPS4	1:4	0.78 ± 0.03	3.33 ± 0.01	1.96 ± 0.01
+ SYUIQ-5	1:2	0.68 ± 0.03	3.35 ± 0.01	1.97 ± 0.03
+ SYUIQ-5	1:3	0.92 ± 0.03	3.26 ± 0.01	1.93 ± 0.03
+ SYUIQ-5	1:4	1.00 ± 0.03*	3.18 ± 0.01	1.91 ± 0.01

*The maximum value is 1; the reported uncertainty comes from the fitting procedure.

### Stacking of G4 units correlates with dimer compactness

The insets in Fig. 4 display Kratky plots of Tel50 in complex with ligands at 1:4 stoichiometric ratio, highlighting the structural changes induced by ligand binding. All curves are consistent with a two-domain system, as evidenced by the presence of two shoulders. Upon ligand binding, the intensity of the shoulder at high-Q decreases compared to that at low-Q. This reduction is minor for BRACO-19 but becomes more pronounced for PhenDC3 and RHPS4, where the plot appears nearly flat between the two signatures. However, the most significant changes are observed for SYUIQ-5, whose Kratky plot shifts toward the bell-shaped profile characteristic of compact globular structures. The observed trend is clearly evident in the overlaid Kratky plots shown in Supplementary Fig. S9. These findings unambiguously suggest that all investigated ligands, to different extents, increase the compactness of the dimer by bringing the two G4 units closer together. The same effect is obtained at lower ligand concentrations, even though to a lesser extent (Supplementary Fig. S10). This result also emerges clearly by inspecting the pair-distance distributions (Supplementary Fig. S11).

**Figure 5.**
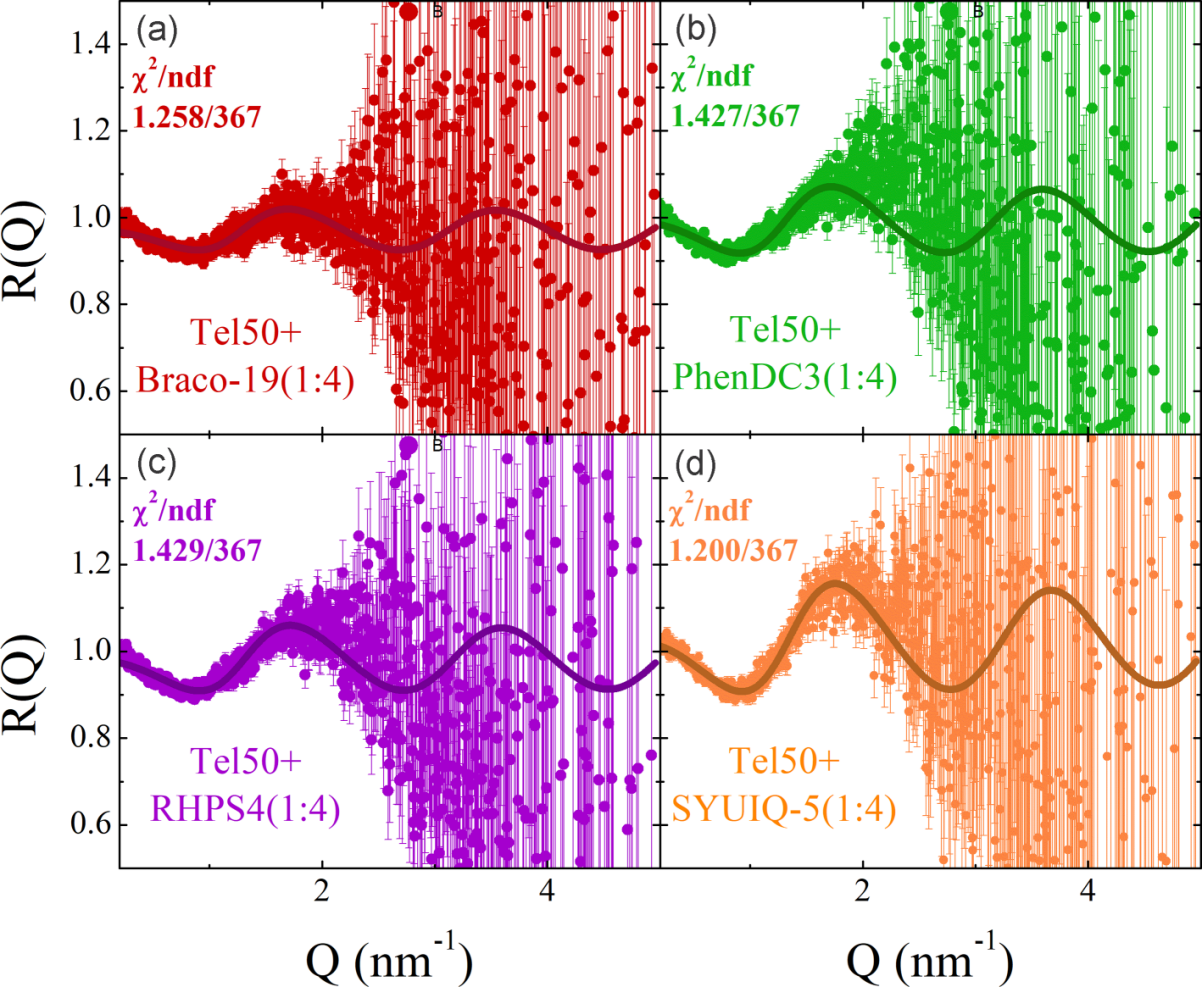
Ratio R(Q) of the SAXS signals of Tel50 in mixture with (**a**) BRACO-19, (**b**) PhenDC3, (**c**) RHPS4, and (**d**) SYUIQ-5 (1:4 DNA/ligand ratio). The best fit using Equation 3 is also reported (solid lines).

**Figure 6.**
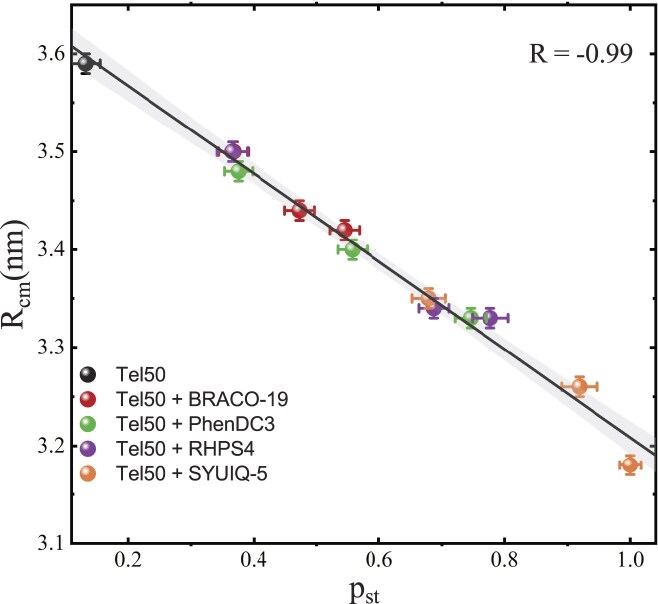
Correlation between the fraction of stacked sites retrieved from the ECG-MC simulations and the effective distance between the centers of mass of the monomeric units obtained by means of Equation (5). For each ligand, increasing stoichiometric ratios correspond to higher values of *p*_st_ and lower values of *R*_cm_. The continuous line is reported to highlight the linear trend.

A quantitative measure of the degree of the dimer compactness is provided by the effective distance between the centers of mass of the G4 units (*R*_cm_), estimated as described in the “*Materials and Methods*” section. The experimental *R*(*Q*) curves, along with the corresponding fit obtained by Equation (5), are reported in Fig. 5 for all the ligands at a 1:4 stoichiometric ratio (see Supplementary Figs S12 and S13 for full dataset). The values *R*_cm_ (Table 1) quantitatively support the picture of the ligand-induced compaction of the dimers. Notably, *R*_cm_ shows a negative correlation with *p*_st_ (Fig. 6), indicating that stronger stacking interactions promote more compact dimeric conformations, bringing the two G4 units closer together on average. This picture was further confirmed by the correlation between *p*_st_ and *R*_g_ (both real and reciprocal space estimates, Supplementary Figs S14 and S15). It is worth noting that, when the majority of junctions are stacked, most of the monomeric units are in proximity, yielding a narrow *R*_cm_ distribution centered at ~3 nm. Conversely, an ensemble of mainly unstacked monomers is characterized by a wider *R*_cm_ distribution, with an additional broad peak at around 3.6 nm (Supplementary Fig. S16).

### Ligands have comparable effects on the topology of monomeric and dimeric G4s

**Figure 7.**
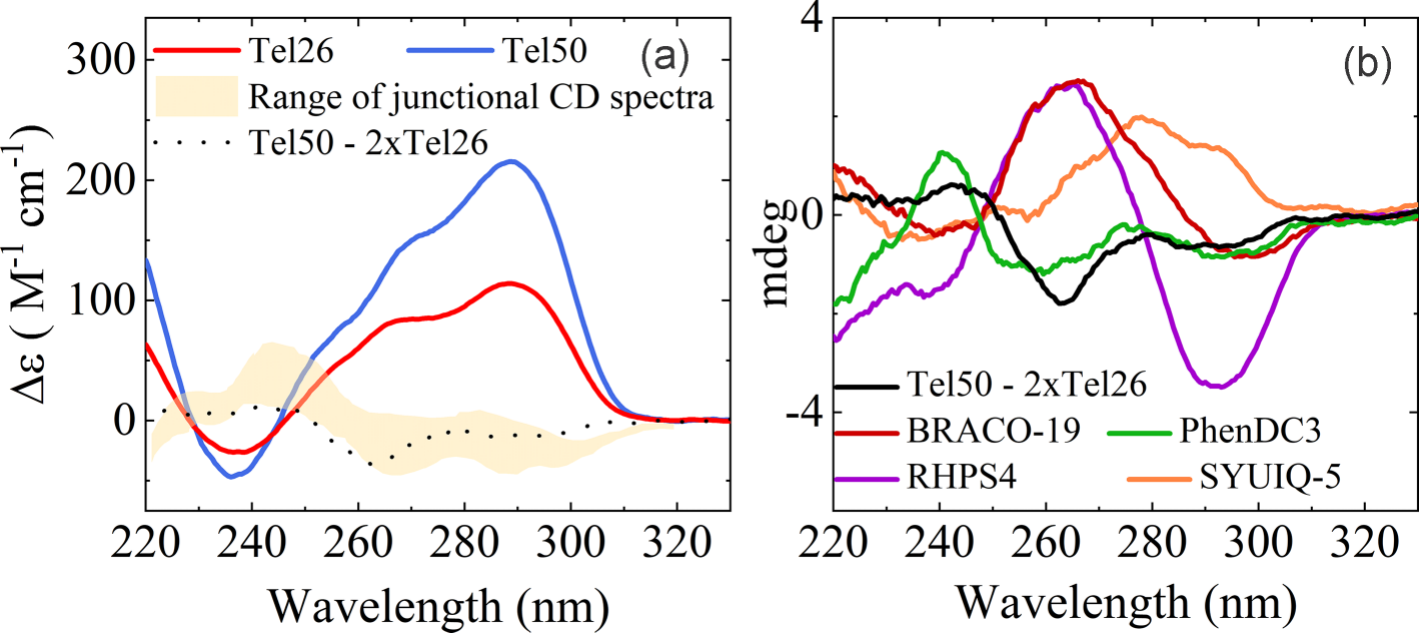
(**a**) Comparison of the CD spectra of Tel26 (red) and Tel50 (blue). The yellow area indicates the range of the “junctional” CD spectrum [49], while the black dashed line represents the difference spectrum obtained by subtracting 2×Tel26 from Tel50. (**b**) Difference spectra of Tel50 and Tel26 in the presence of ligands, with BRACO-19 (red), PhenDC3 (green), RHPS4 (purple), and SYUIQ-5 (orange).

CD spectroscopy was employed to characterize the secondary structures of Tel26 and Tel50, which are expected to predominantly adopt hybrid G4 topologies [51]. The CD spectra show a maximum around 290 nm, a shoulder at 270 nm, and a minimum at 240 nm (Fig. 7a). As previously reported, the spectra of higher-order telomeric G4s cannot be reconstructed by a simple sum of the contributions of individual monomeric units, as a significant contribution arises from interactions between contiguous G4 units [49].

To investigate these effects, we computed the difference spectrum between Tel50 and Tel26 normalized to the number of G4 units. This analysis emphasizes the structural distinctions between the dimer and the monomer, capturing a junctional signal arising from stacking interactions between G4 units and additional inter-unit interactions that constrain the loop regions [49, 52]. Notably, the resulting spectrum falls within the range of the previously characterized “junctional” signal (yellow area in Fig. 7a), as observed for the Tel48 sequence [49], suggesting that the monomers of Tel50 and Tel26 share a highly similar overall topology.

In addition to CD, G4 formation in both Tel26 and Tel50 was validated by TDS and 1D ¹H NMR experiments. The TDS profiles displayed the expected G4 signature, with a negative band at 295 nm and positive bands at 273 and 243 nm (Supplementary Fig. S17). The ¹H NMR spectra exhibited imino proton signals in the 10.4–12.6 ppm region, corresponding to guanine imino protons involved in Hoogsteen hydrogen bonding (Supplementary Fig. S18). Notably, the ¹H NMR spectrum of Tel50 differs from that of Tel26, reflecting its greater structural complexity. Complementary native PAGE analysis (Supplementary Fig. S5) revealed that Tel26 migrates with mobility comparable to mTel24 (a monomeric G4 used as a control), consistent with a single G4 species. In contrast, Tel50 exhibits reduced mobility, as expected for a species with approximately twice the molecular weight, and shows stronger GelRed staining, indicative of increased secondary structure content. Together, these results confirm G4 formation in both sequences and support the presence of two tandem G4 units in the longer Tel50 sequence, in agreement with previous studies [19, 49, 53, 54, 55].

To explore how ligands interact with these G4 structures, we next compared the CD difference spectra of Tel50 and Tel26 in the presence of ligands at the highest stoichiometric ratios (DNA-to-ligand 1:2 and 1:4 for Tel26 and Tel50, respectively) (Fig. 7b). The difference spectrum between Tel50 + PhenDC3 (1:4 ratio) and Tel26 + PhenDC3 (1:2 ratio) closely resembles the one obtained without ligands (green versus black line in Fig. 7b). This observation suggests that PhenDC3 binding exerts only a minor influence on the inter-G4 linker and that the ligand-induced structural rearrangements within the dimer largely mirror those observed for the monomer.

In contrast, binding of the telomeric sequences to BRACO-19, RHPS4, and SYUIQ-5 yields difference spectra that deviate markedly from the ligand-free profiles, likely reflecting DNA–ligand interactions that differentially perturb the monomeric and dimeric forms.

**Figure 8.**
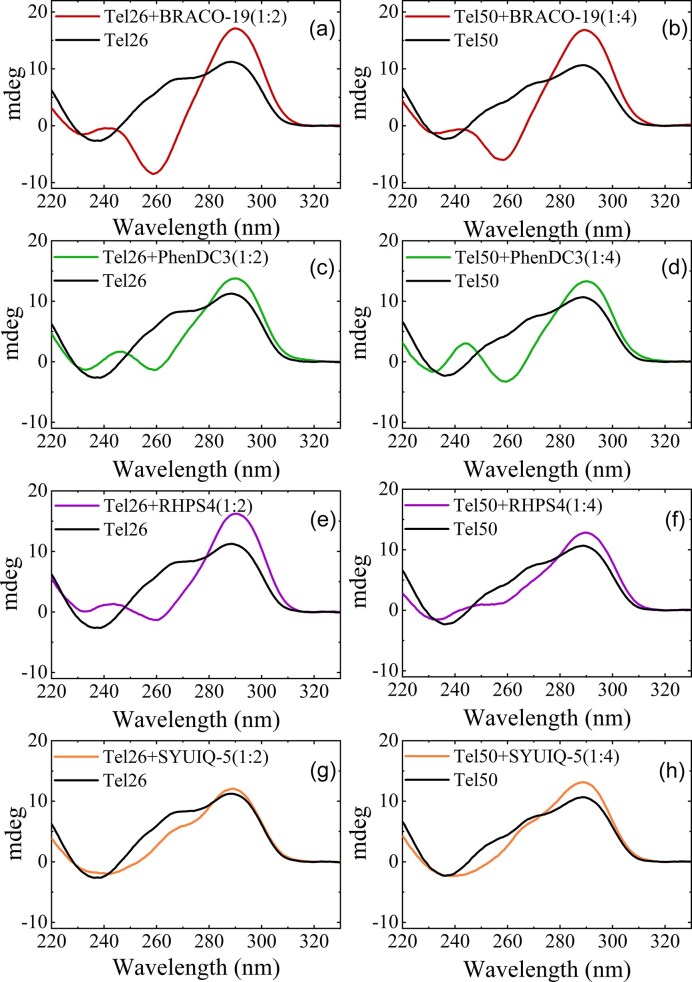
CD profiles of Tel26 and Tel50 in the absence (black lines) and presence (colored lines) of ligands: (**a**) Tel26 + BRACO-19 (1:2, red). (**b**) Tel50 + BRACO-19 (1:4, red). (**c**) Tel26 + PhenDC3 (1:2, green). (**d**) Tel50 + PhenDC3 (1:4, green). (**e**) Tel26 + RHPS4 (1:2, purple). (**f**) Tel50 + RHPS4 (1:4, purple). (**g**) Tel26 + SYUIQ-5 (1:2, orange). (**h**) Tel50 + SYUIQ-5 (1:4, orange).

Figure 8 shows the CD profiles of Tel26 and Tel50 in the absence and presence of ligands at the highest stoichiometric ratios (DNA-to-ligand 1:2 and 1:4 for Tel26 and Tel50, respectively), while the ligand-dependent changes across intermediate ratios are reported in Supplementary Figs S19 and S20. Overall, the ligands induce structural rearrangements in the Tel50 dimer that closely match those observed for the Tel26 monomer. BRACO-19 and PhenDC3 both promote an antiparallel-like G4 conformation, characterized by a maximum at 290 nm, a minimum near 260 nm, and an additional maximum around 240 nm. Notably, the 260 nm minimum is generally less pronounced in Tel50 than in Tel26. RHPS4 induces a comparable response, but with notable differences: it enhances the 290 nm maximum, while the minimum at 260 nm is less intense than with BRACO-19 and PhenDC3, particularly in Tel50, suggesting an interconversion between distinct hybrid forms. SYUIQ-5 perturbs the hybrid topology to an even lesser extent, likely reflecting a more limited shift in the equilibrium between hybrid conformations [56].

### Ligands induce comparable thermal stabilization in monomeric and dimeric G4s

**Figure 9.**
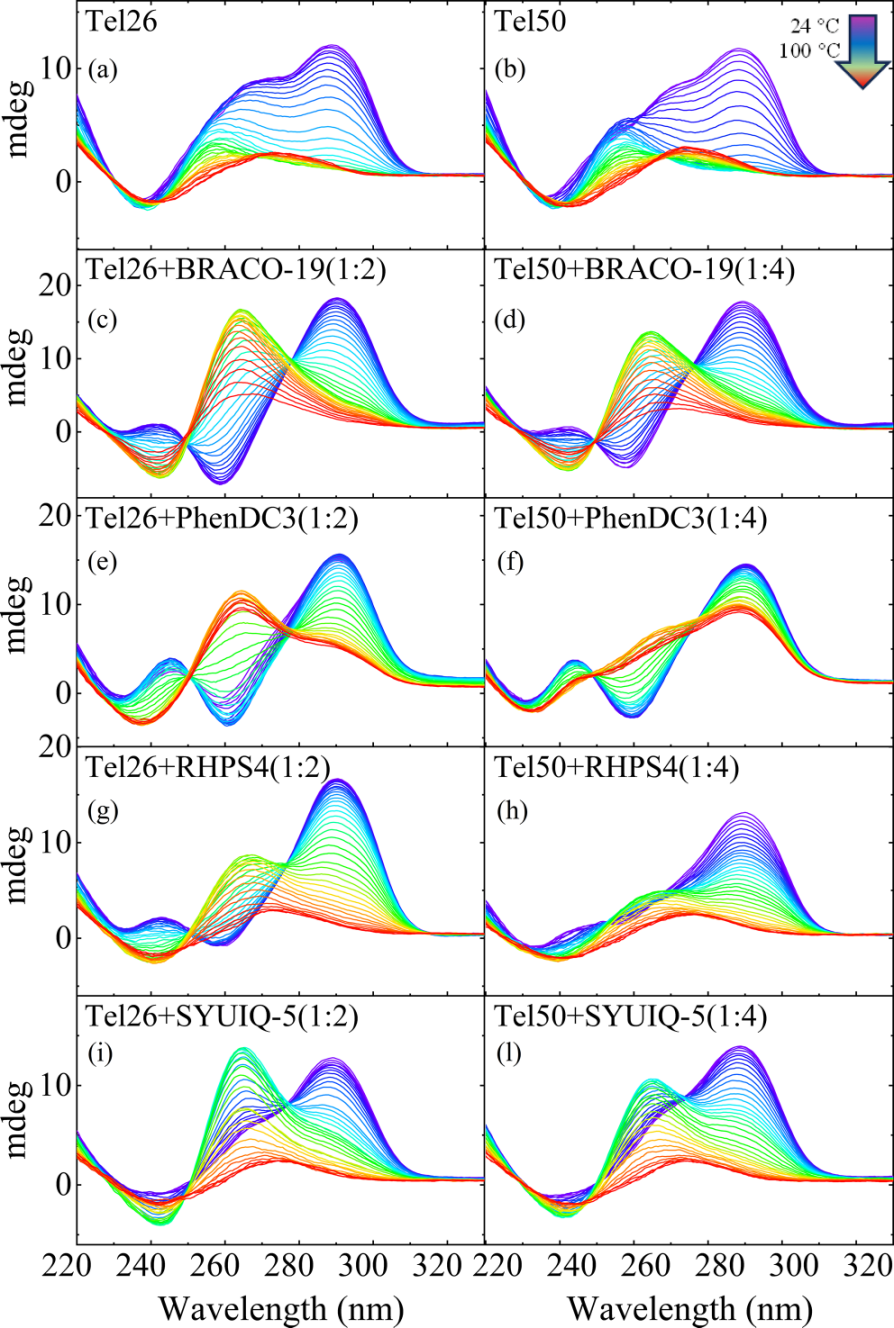
CD melting spectra of Tel26 and Tel50 in the absence and presence of ligands (DNA-to-ligand ratios of 1:2 for Tel26 and 1:4 for Tel50).

Temperature-dependent CD experiments were performed to study the effects of ligands on the thermal stability of G4s formed by Tel26 and Tel50 (Fig. 9 and Supplementary Figs S21–S23). Since G4 unfolding typically proceeds through intermediate states [57–60], we applied SVD to characterize the melting processes. Tel26 and Tel50 display similar melting temperatures, within experimental error (∼62°C; Supplementary Tables S3 and S4). SVD analysis (Supplementary Fig. S24) indicates that both samples follow a three-state unfolding pathway with a single intermediate.

Upon heating, all ligands induced marked spectral changes (Fig. 9 and Supplementary Figs S21–S23), characterized by the appearance of a strong positive band at ∼260 nm and a more intense minimum around 240 nm (especially for BRACO-19 and SYUIQ-5), along with the decrease in the intensity of the 290 nm band. Such spectral features are indicative of the formation of an intermediate state whose profile closely resembles that of a parallel-stranded G4 or G-triplex [11, 61]. However, the extent of this transition, as well as the temperature at which it appears, varies across ligands. SVD analysis further elucidated the thermal evolution of species, confirming the presence of at least one intermediate in all DNA–ligand complexes (Supplementary Figs S25–S28). All ligands showed a pronounced stabilizing effect on the G4 structures, in both monomeric and dimeric forms. Supplementary Tables S3 and S4 show *T*_m_ values (red). While the RHPS4 and SYUIQ-5 complexes undergo complete unfolding, BRACO-19 and PhenDC3 stabilize the sequences to a greater extent. In particular, in the presence of BRACO-19, at the highest stoichiometric ratio, the CD spectra of Tel26 and Tel50 retained the characteristic signatures of folded structures even at the highest temperatures tested (T_m_ > 90°C). PhenDC3 displays distinctive behavior. At low ligand concentrations (Tel26 + PhenDC3 1:1 and Tel50 + PhenDC3 1:2, Supplementary Figs S21 and S22), the G4 structure predominantly retains a hybrid topology, gradually shifting toward a parallel-like conformation at high temperature. At higher DNA-to-ligand ratios, Tel26 + PhenDC3 1:2 and Tel50 + PhenDC3 1:4 (Fig. 9e and f) have different thermal responses. Starting from the ligand-induced antiparallel topology at low temperature, Tel26 adopted a parallel-like conformation upon heating, whereas Tel50’s antiparallel state progressively evolved toward a hybrid-like form. This concentration-dependent behavior is also observed in the PhenDC3-induced G4 stabilization: at the highest ligand ratios, PhenDC3 was found to be the most effective stabilizer for both sequences, with CD spectra indicating highly structured species even at 100°C (Fig. 9e and f). Conversely, at the lowest ratios, PhenDC3 does not stabilize the structures of Tel26 and Tel50. However, for Tel26 + PhenDC3 (1:1) and Tel50 + PhenDC3 (1:2), the CD profile at high temperatures does not become completely featureless. This may indicate the presence of weak residual base-stacking interactions, resulting in a residual structured and slightly more intense CD signal compared with the other samples, even though the unfolding process has effectively occurred.

## Discussion

In this study, we combined CD spectroscopy and SAXS measurements with ECG-MC simulations to investigate the effects of well-established ligands on G4-forming human telomeric sequences. We focused on the higher-order dimeric G4, Tel50, consisting of two tandem G4 units, and compared it with its monomeric counterpart, Tel26, enabling a multiscale analysis of ligand interactions with both monomeric and dimeric G4 architectures. SAXS data, supported by ECG-MC simulations, show that ligand binding consistently promotes stacking of G4 units within the dimer. CD analysis, complemented by the SVD method, revealed that despite the stacked arrangement, ligand-induced thermal stabilization of G4 dimers is comparable to that of their monomeric analogues.

Previous investigations have reported that certain ligands, such as oxazole–triazoles [62], triaryl-substituted imidazole derivatives (e.g. IZNP-1) [22], square-planar Pt(II) salphens [63], quinoline-based ligands [64], and cationic porphyrin derivatives [65], exhibit enhanced affinity, selectivity, and thermal stabilization toward human telomeric G4 dimers compared to monomers. This behavior has typically been attributed to ligand intercalation or end‐stacking at the G4–G4 interface, accompanied by additional π–π stacking and electrostatic interactions that stabilize the dimeric assembly [19, 22, 62, 65]. In contrast, other molecules such as naphthalene diimides have been shown to stabilize monomers and dimers to a similar extent [19, 66]. Notably, some ligands even induce partial destabilization of G4 dimers, as observed for the cationic porphyrin derivative p-TMPipEOPP [67], the triazatruxene derivative AZATRUX [21], and the RHAU peptide [68]. These destabilizing effects likely arise from less favorable interactions or steric hindrance at the dimer interface, leading to reduced stabilization of G4 dimers compared to their monomeric counterparts.

The molecules investigated in this study, i.e. BRACO-19, PhenDC3, RHPS4, and SYUIQ-5, appear to fall into the second category. The nearly identical melting temperatures observed for Tel26 (monomer) and Tel50 (dimer) at equivalent molar ratios relative to G4 support the fact that these ligands predominantly interact with a single G4 unit rather than bridging the two structural motifs.

Although none of the four ligands establish strong bridging interactions at the G4–G4 junction, all promote the formation of more compact complexes compared to the unbound dimers. The structural features of each compound may provide a rationale for this effect. SYUIQ-5, an indoloquinoline ligand substituted at C-11 with a positively charged nitrogen atom, capable of engaging in both π–π stacking and electrostatic interactions [69, 70], proved to be the most effective in inducing compactness. This likely arises from its small, wedge-shaped scaffold, which fits precisely into the narrow G4–G4 interface, making it particularly suited for compacting the dimeric structure. RHPS4, a pentacyclic acridinium, has been shown to interact in a similar manner at the terminal G-quartets of hybrid G4 structures, favoring closer stacking of domains [71]. PhenDC3 appears to be predominantly characterized by its intercalative binding mode [72]. In both Tel26 and Tel50, complexation with PhenDC3 induces secondary structure conformational changes like those observed in Tel23, where ligand intercalation promotes a transition from a hybrid to an antiparallel topology [73]. Finally, BRACO-19 has been reported to interact via top or bottom stacking depending on G4 topology [74]. Nevertheless, its bulky side chains may impose steric constraints that hinder insertion into narrow interfacial pockets, explaining its limited ability to compact the dimer. These results suggest that ligand structure, rigidity, and preferred binding mode(s) play crucial roles in promoting the formation of more compact dimers.

To quantify the spectral differences between the topologies of complexed and free dimers, we calculated the RSQ quantity between the respective CD curves (from Fig. 8 and Supplementary Table S5). This analysis provides a semi-quantitative metric of conformational differences as captured by CD spectroscopy (Table 2). In the investigated samples, the higher the RSQ, the larger the topology change of dimers (or monomers) upon complexation. The RSQ values suggest that ligands induce a larger change of topology on monomers than dimers at the highest molar ratio. This is probably because in the dimer the G-tetrads delimiting the junctional pocket are less accessible to ligands, thus limiting the possible end-stacking binding modes on the G4 units. Strikingly, combined RSQ and stacking fraction (*p*st) data demonstrate that large ligand-induced conformational changes are not a prerequisite for dimer compaction. Consistent with this, BRACO-19 exhibits the highest RSQ yet the smallest compaction effect, while SYUIQ-5 drives efficient dimer compaction in the absence of extensive structural rearrangement. Interestingly, we notice that the smallest ligand that minimizes steric clashes can promote compaction without major conformational remodeling.

**Table 2.** RSQ between the CD curves of the complexes and those of the telomers alone

Sample		RSQ (mdeg)
BRACO-19	Tel50 (4:1)	64.931
BRACO-19	Tel26 (2:1)	83.004
PhenDC3	Tel50 (4:1)	50.191
PhenDC3	Tel26 (2:1)	50.451
RHPS4	Tel50 (4:1)	26.475
RHPS4	Tel26 (2:1)	51.977
SYUIQ-5	Tel50 (4:1)	22.054
SYUIQ-5	Tel26 (2:1)	23.823

Our results further suggest that larger conformational changes, rather than increased compactness, relate to stronger thermal stabilization. Indeed, at higher stoichiometric ratios (1:4), the more thermally stable complexes, Tel50 + BRACO-19 and Tel50 + PhenDC3, which remain partially folded at 90°C, are associated with higher RSQ values. In contrast, Tel50 + SYUIQ-5 and Tel50 + RHPS4 (1:4), which have lower melting temperatures, exhibit lower RSQ values. Furthermore, thermal stability appears to depend mainly on the ligand’s binding mode to the monomeric unit and the resulting structural rearrangements, whereas stacking between G4 units does not necessarily confer additional stabilization.

## Conclusions

The combined approach applied here to study telomeric G4 monomers and dimers complexed with well-established, high-affinity ligands of distinct structures and chemical properties shows that all investigated compounds promote the formation of more compact dimeric assemblies. SAXS analyses reveal global compaction of the Tel50 constructs, whereas CD measurements indicate ligand-dependent changes in G4 topology. SAXS-guided ECG-MC simulations, while not capturing these topological differences at the atomistic level, provide an efficient quantitative description of stacking behavior and ligand–G4 interactions, as well as insights into changes in the quaternary organization of the complexes, a structural level to which SAXS is particularly sensitive, thereby supporting multiscale characterization. Globally, the various techniques suggest that ligand-induced stacking contributes to dimer compaction, although the interactions largely remain localized to individual G4 units. As a result, thermal stabilization does not correlate simply with global compactness, but is mainly determined by the ligand binding mode at each G4 unit and the associated conformational rearrangements. While the present approach, combining complementary techniques, has the potential to evolve into a fully integrated structural framework, achieving this goal will require a systematic investigation of the capability of SAXS–WAXS methods to resolve G4 topological features, an area that remains largely unexplored. Nevertheless, the results reported here represent an important step forward, revealing an intricate balance between ligand-induced structural reorganization, complex stability, and dimer compactness in multimeric G4 systems. Overall, these findings emphasize the importance of considering both G4 topology and ligand structure in the rational design of G4-targeting molecules with improved selectivity and efficacy.

## Supplementary Material

gkag403_Supplemental_File

## Data Availability

All the supporting data of this study are available within the article or through the supplementary data files. The data analysis scripts have been deposited in Zenodo at https://doi.org/10.5281/zenodo.19499271. SASBDB entry numbers (https://www.sasbdb.org/). SASDY63 – human telomeric g-quadruplex (Tel50), SASDY73 – human telomeric g-quadruplex (Tel26), SASDYA3 – human telomeric g-quadruplex Tel50+BRACO-19 (1:4) SASDYB3 – human telomeric g-quadruplex Tel50+PhenDC3 (1:2), SASDYC3 – human telomeric g-quadruplex Tel50+PhenDC3 (1:3), SASDYD3 – human telomeric g-quadruplex Tel50+PhenDC3 (1:4), SASDYE3 – human telomeric g-quadruplex Tel50+RHPS4(1:2), SASDYF3 – human telomeric g-quadruplex Tel50+RHPS4(1:3), SASDYG3 – human telomeric g-quadruplex Tel50+RHPS4(1:4), SASDYH3 – human telomeric g-quadruplex Tel50+SYUIQ-5 (1:2), SASDYJ3 – human telomeric g-quadruplex Tel50+SYUIQ-5 (1:3), SASDYK3 – human telomeric g-quadruplex Tel50+SYUIQ-5 (1:4), SASDYL3 – human telomeric g-quadruplex Tel26+BRACO-19 (1:1), SASDYM3 – human telomeric g-quadruplex Tel26+BRACO-19 (1:2), SASDYN3 – human telomeric g-quadruplex Tel26+PhenDC3 (1:1), SASDYP3 – human telomeric g-quadruplex Tel26+RHPS4 (1:1), SASDYQ3 – human telomeric g-quadruplex Tel26+PhenDC3 (1:2), SASDYR3 – human telomeric g-quadruplex Tel26+RHPS4 (1:2), SASDYS3 – human telomeric g-quadruplex Tel26+SYUIQ-5 (1:1), SASDYT3 – human telomeric g-quadruplex Tel26+SYUIQ-5 (1:2).
