# Supplementary material for: Ligands reshape the compactness, stability, and topology of telomeric G-quadruplex dimers
Source: Nucleic Acids Res. 2026 May 4;54(8):gkag403. doi: 10.1093/nar/gkag403 (PMC13136904; doi:10.1093/nar/gkag403)
Supplement: gkag403_Supplemental_File [file gkag403_supplemental_file.pdf]

Supplementary Information

Ligands Reshape the Compactness, Stability, and Topology of Telomeric G-Quadruplex Dimers

Luca Bertini[1,†], Valeria Libera[1,†], Valentina Arciuolo[2], Mattia Trapella[1], Simona Marzano[2], Deniz Mostarac[3], Giorgio Schirò[4], Caterina Petrillo[1], Concetta Giancola[2], Cristiano De Michele[3], Jussara Amato[2], Lucia Comez[5,*], Bruno Pagano[2,*], Alessandro Paciaroni[1,*]

[1]Dipartimento di Fisica e Geologia, Università degli Studi di Perugia, Via Alessandro Pascoli, 06123 Perugia, Italy

[2]Department of Pharmacy, University of Naples Federico II, 80131 Naples, Italy

[3]Dipartimento di Fisica, Università di Roma La Sapienza, Piazzale Aldo Moro 2, 00185 Roma, Italy

[4]CNRS, Institut de Biologie Structurale, 71 Avenue des Martyrs, 38044 Grenoble, France

[5]CNR-Istituto Officina dei Materiali (IOM), Unità Perugia, 06123 Perugia, Italy

FIGURES

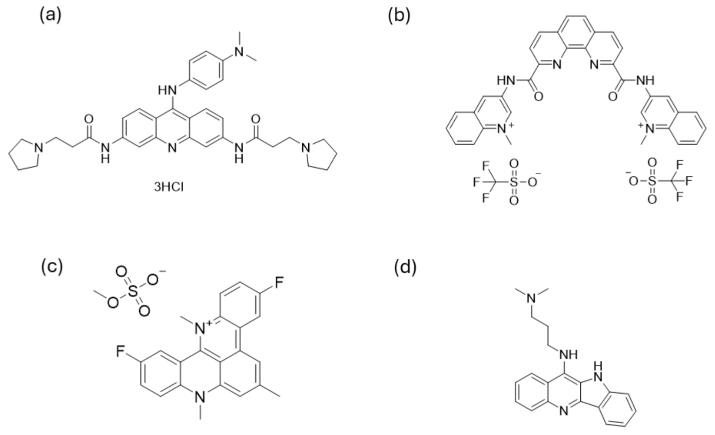

Figure S1. Chemical structures of (a) BRACO-19, (b) PhenDC3, (c) RHPS4, and (d) SYUIQ-5.

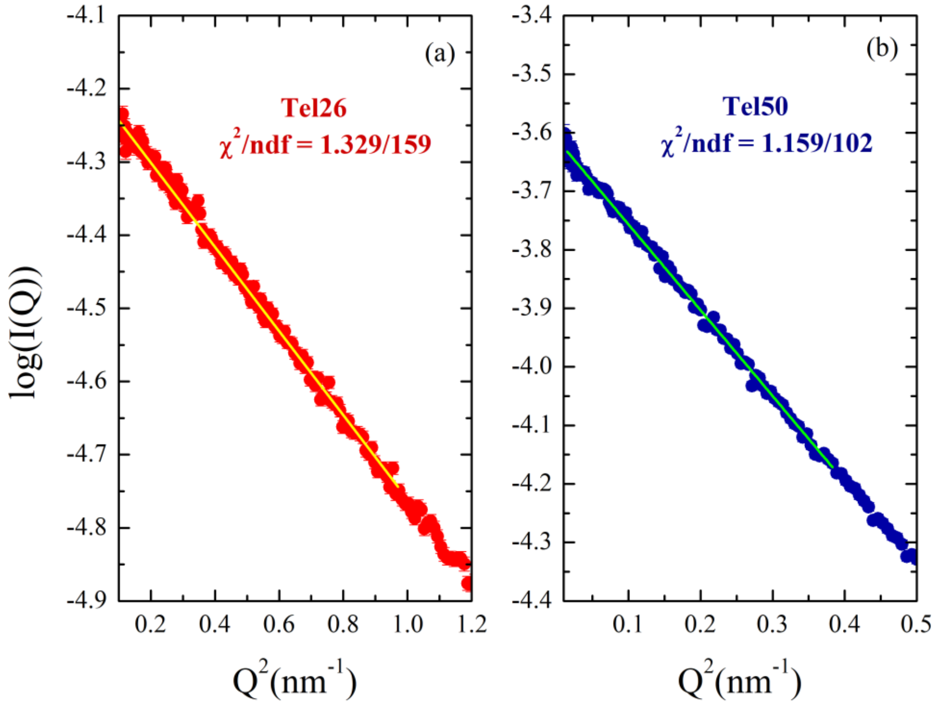

Figure S2. Guinier fit obtained from the AutoRg routine[1] for (a) Tel26 and (b) Tel50. The reduced chi-squared reported in the legend is the one obtained by a single Guinier fit on the optimal range detected by running AUTORG.

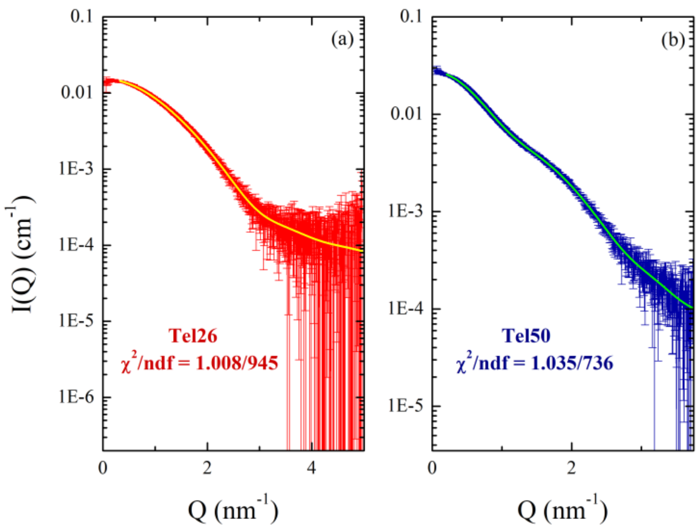

Figure S3. Best fitting curves used to reconstruct the p(r) distribution of (a) Tel26 and (b) Tel50.

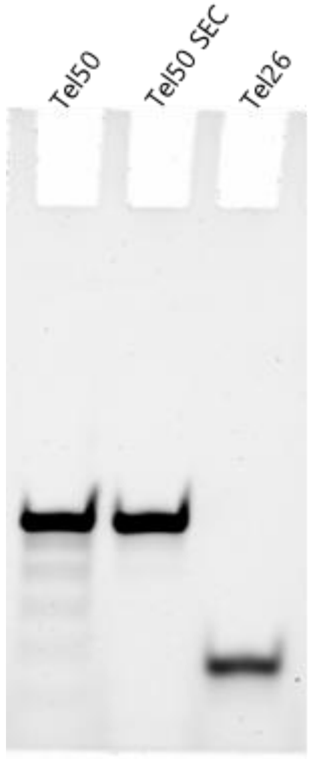

Figure S4. Native polyacrylamide gel electrophoresis for Tel50 (before and after SEC) and Tel26. Oligonucleotide samples were loaded at 5 µM concentration in 5 mM KH_2PO_4/K_2HPO_4 buffer at pH 7.0, supplemented with 20 mM KCl and resolved on 12.5% native PAGE. Bands were visualized by using GelRed® nucleic acid stain.

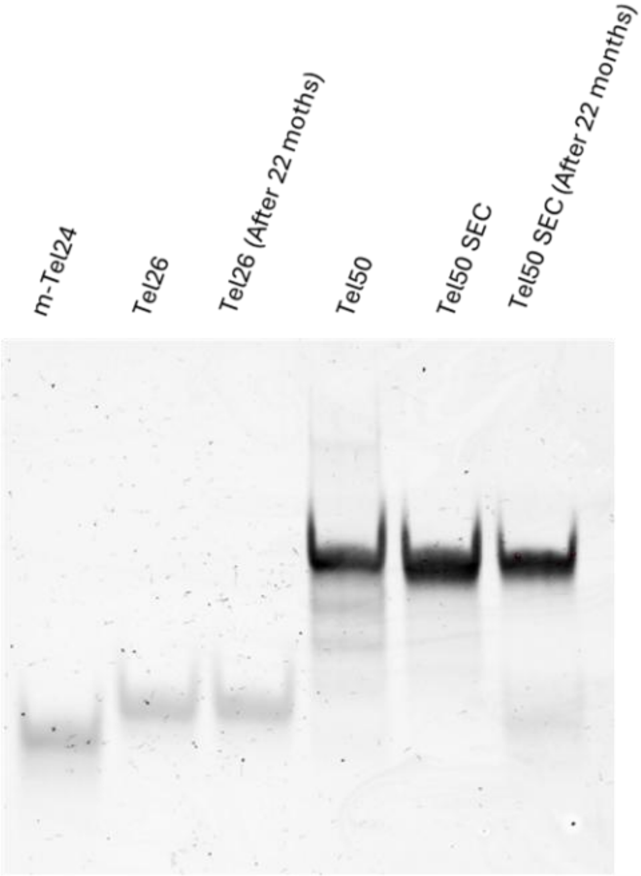

Figure S5. Native polyacrylamide gel electrophoresis (PAGE) for m-Tel24, Tel26 and Tel50. Oligonucleotide samples were loaded at 5 μM concentration in 5 mM KH_2PO_4/K_2HPO_4 buffer at pH 7.0, supplemented with 20 mM KCl and resolved on 12.5% native PAGE. Bands were visualized by using GelRed® nucleic acid stain.

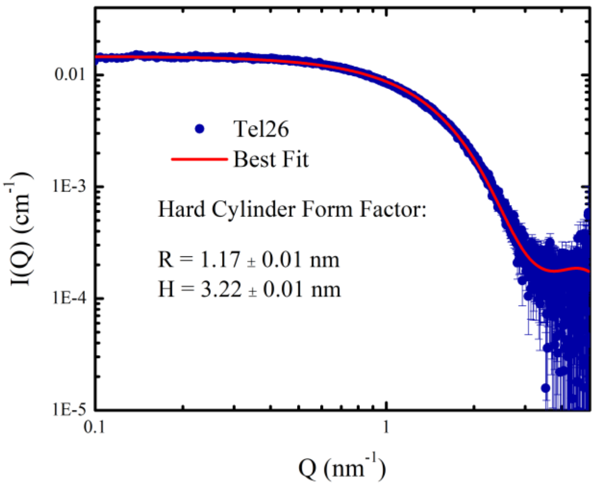

Figure S6. SAXS signal of Tel26 (blue) along with the best fitting HC form factor (red line).

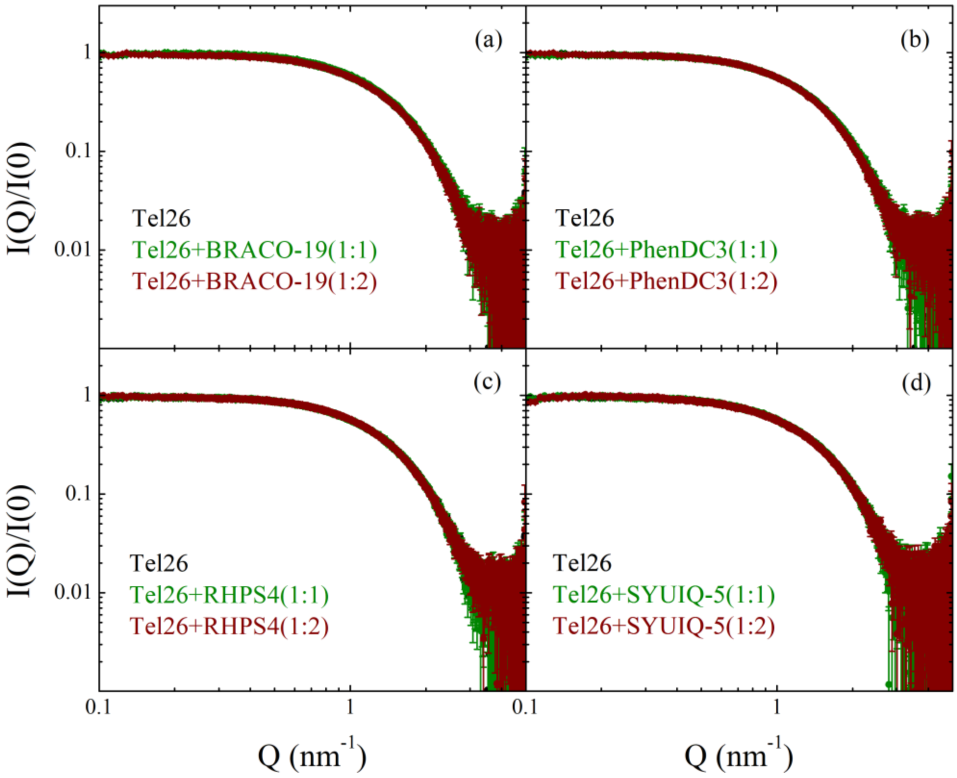

Figure S7. SAXS signals of Tel26 (black curves) and Tel26 + BRACO-19 (a), Tel26 + PhenDC3 (b), Tel26 + RHPS4 (c) and Tel26 + SYUIQ-5 (d). The molar stoichiometric ratios 1:1 (red) and 1:2 (red) are reported.

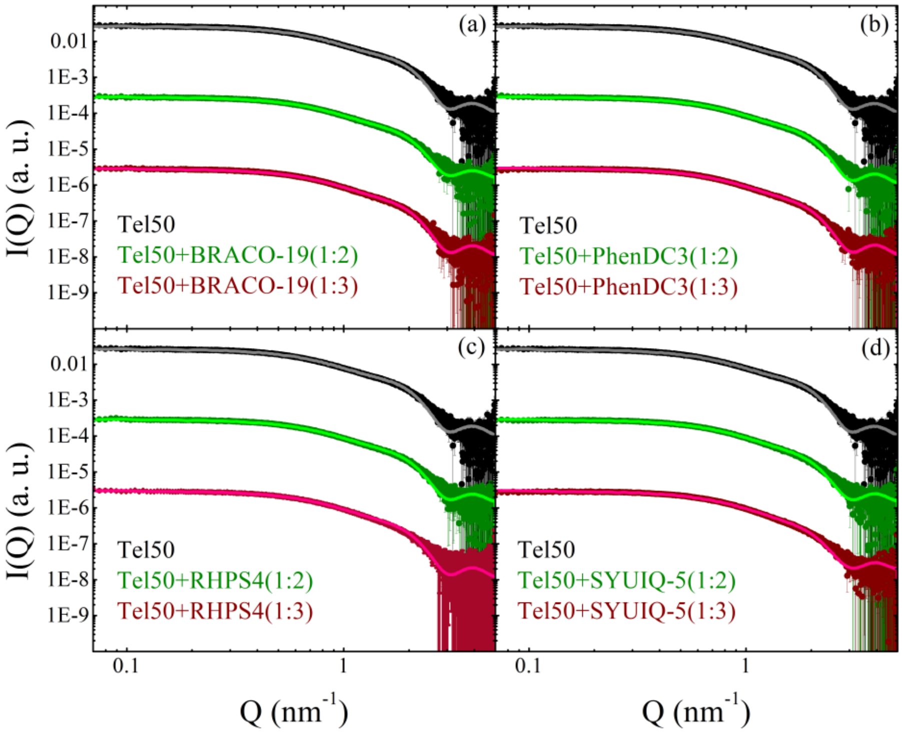

Figure S8. SAXS signals of free Tel50 (black curves) and Tel50 + ligands (at 1:2 and 1:3 stoichiometric ratios): (a) Tel50 + BRACO-19, (b) Tel50 + PhenDC3, (c) Tel50 + RHPS4, and (d) Tel50 + SYUIQ-5. Experimental curves for the 1:2 (green) and 1:3 (red) complexes are shown together with the reconstructed SAXS intensities obtained from the best fitting ECG simulations (gray, light green and light red curves, respectively).

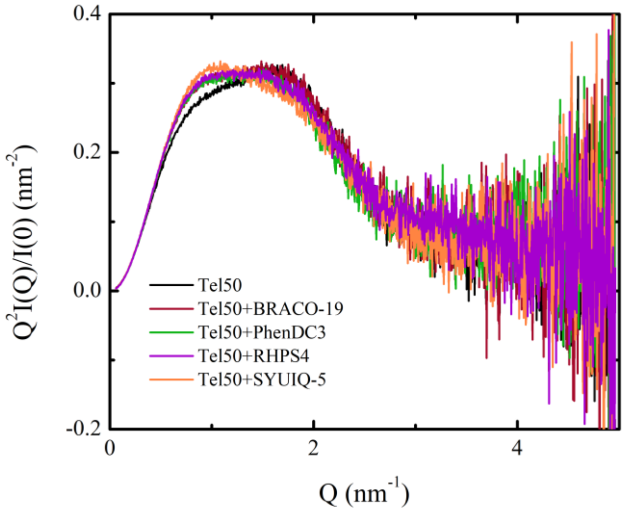

Figure S9. Kratky plots of Tel50 and Tel50+ligands at the highest stoichiometric ratio (1:4).

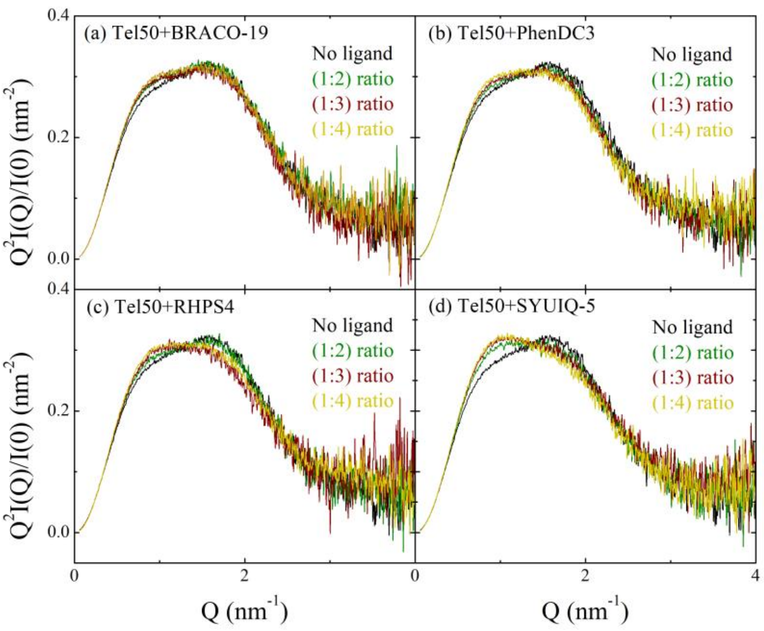

Figure S10. Kratky plots of Tel50 + BRACO-19 (a), Tel50 + PhenDC3 (b), Tel50 + RHPS4 (c) and Tel50 + SYUIQ-5 (d). Uncomplexed Tel50 (black) is reported along with the molar stoichiometric ratios 1:2 (green), 1:3 (red), and 1:4 (yellow).

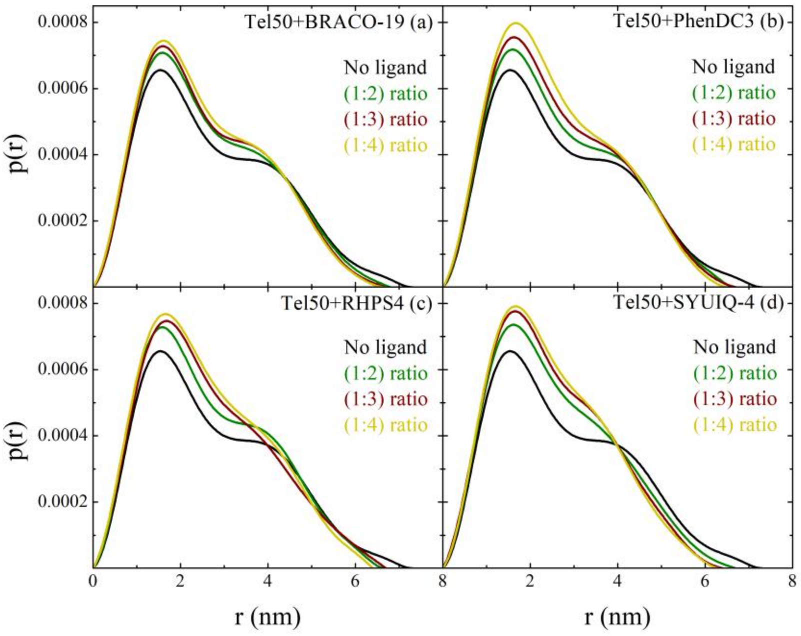

Figure S11. Pair-distance distributions of Tel50 + BRACO-19 (a), Tel50 + PhenDC3 (b), Tel50 + RHPS4 (c) and Tel50 + SYUIQ-5 (d). Uncomplexed Tel50 (black) is reported along with the molar stoichiometric ratios 1:2 (green), 1:3 (red), and 1:4 (yellow).

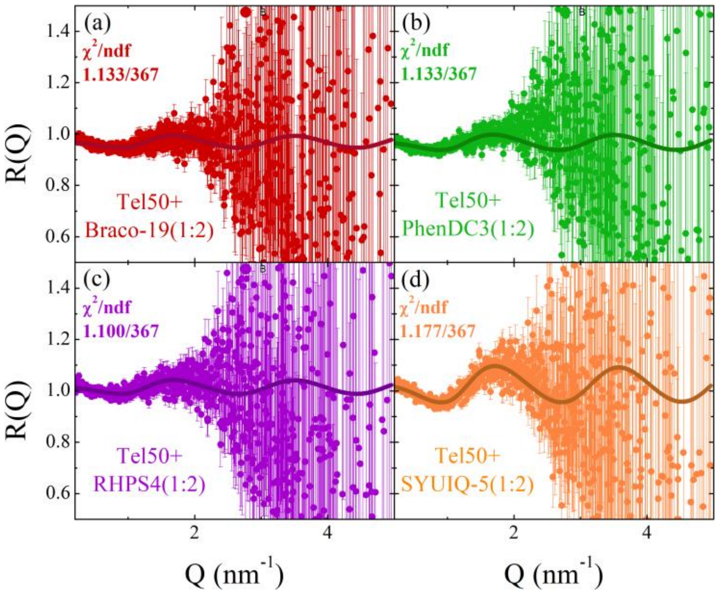

Figure S12. R(Q) of Tel50 + BRACO-19 (a), Tel50 + PhenDC3 (b), Tel50 + RHPS4 (c), and Tel50 + SYUIQ-5 (d) at the molar stoichiometric ratio 1:2, along with the corresponding best fit obtained using Eq. 5.

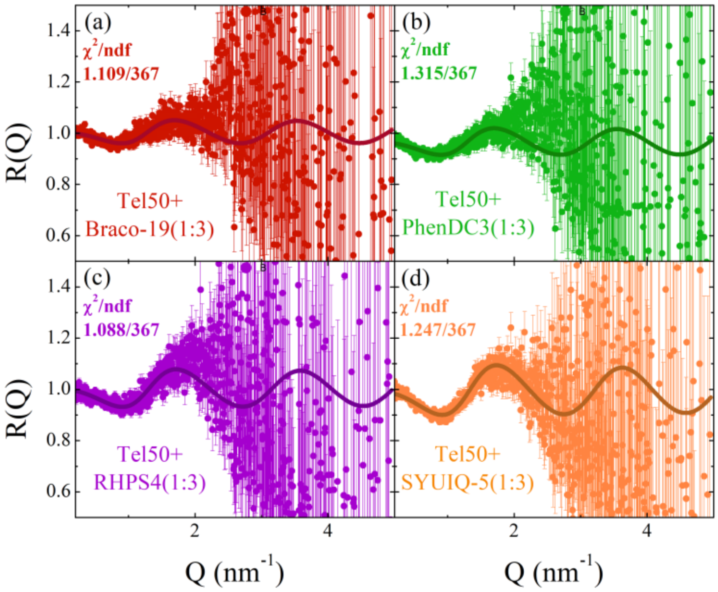

Figure S13. R(Q) of Tel50 + BRACO19 (a), Tel50 + PhenDC3 (b), Tel50 + RHPS4 (c), and Tel50 + SYUIQ-5 (d) at the molar stoichiometric ratio 1:3, along with the corresponding best fit obtained using Eq. 5.

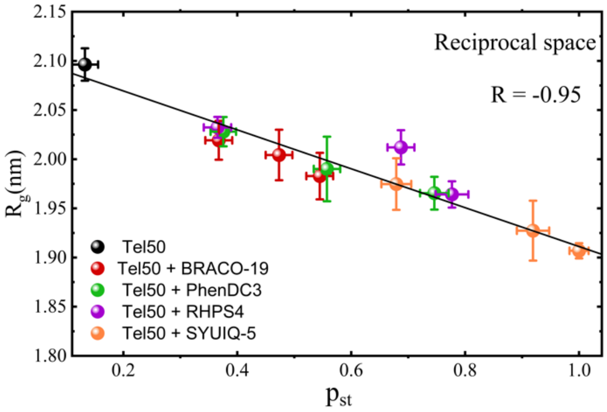

Figure S14. Correlation between the fraction of stacked sites retrieved from the ECG Simulations and the radius of gyration obtained by using the AutoRg routine on the SAXS signals (Eq. S1). For each ligand, increasing stoichiometric ratios correspond to higher values of p_{st} and lower values of R_g.

$$I(q) = I(0)e^{-(q^2 R_g^2/3)} \qquad \text{Eq. S1}$$

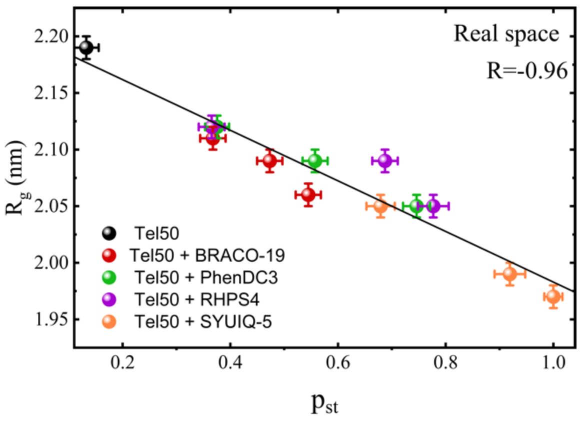

Figure S15. Correlation between the fraction of stacked sites retrieved from the ECG Simulations and the radius of gyration obtained from the p(r) distribution computed from the SAXS signals (Eq. S2). For each ligand, increasing stoichiometric ratios correspond to higher values of p_{st} and lower values of R_g.

$$R_g^2 = \frac{\int_0^{D_{max}} r^2 p(r) dr}{\int_0^{D_{max}} p(r) dr} \qquad \text{Eq. S2}$$

with D_{max} is defined as the distance at which *p(r)* goes to zero[2].

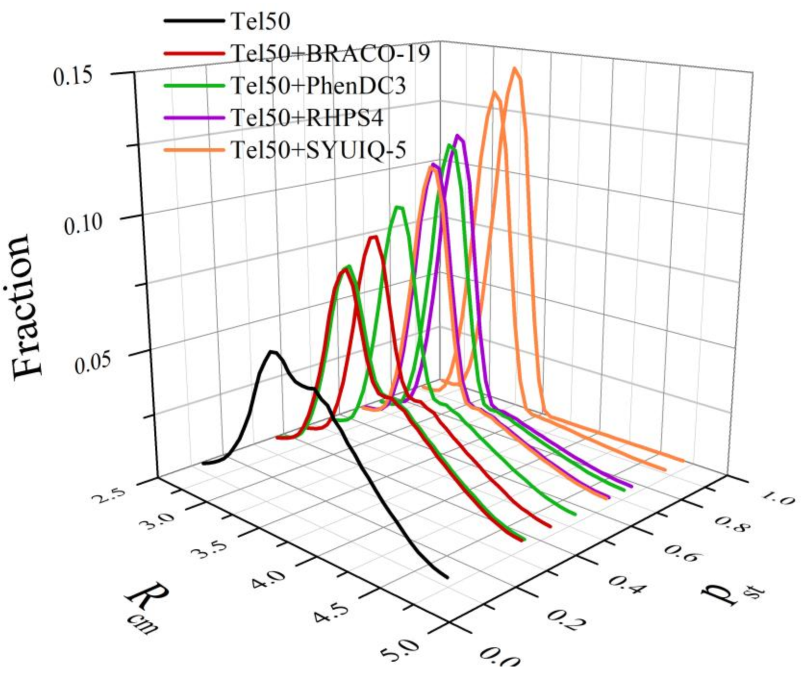

Figure S16. Distribution of the distance between the centers of mass of the two hard cylinder units, obtained from the best-fitting ECG simulations. The overall distribution is the combination of two components: one for the stacked configurations (narrow peak at R_{cm} ~ 3 nm) and another for the unstacked configurations (wider distribution centered at R_{cm} ~ 3.6 nm), weighted by p_{st} and $1-p_{st}$, respectively. Increasing molar stoichiometric ratios correspond to a higher weight for the distribution corresponding to stacked configurations. The samples Tel50 + BRACO-19 (1:2) and Tel50 + RHPS4 (1:2) are superimposed.

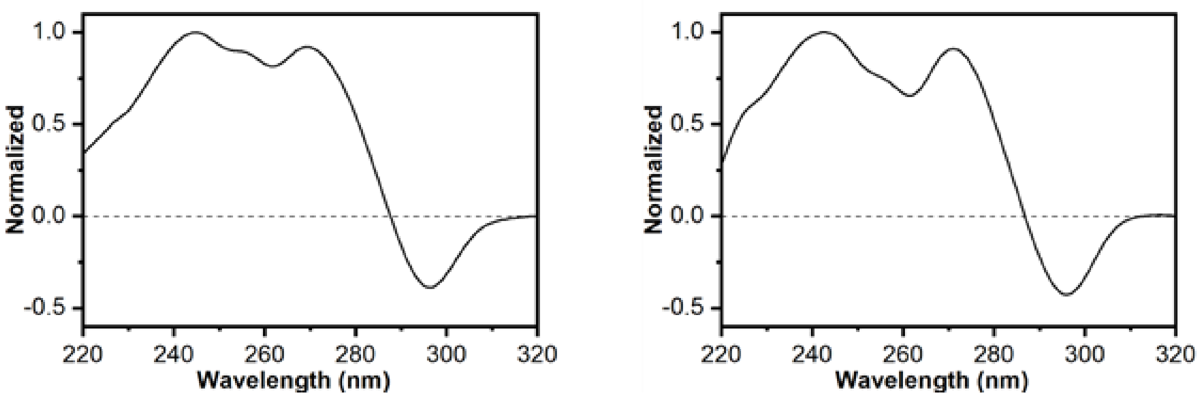

Figure S17. Thermal difference spectra (TDS) of Tel26 (left) and Tel50 (right) in 5 mM KH_2PO_4/K_2HPO_4 buffer (pH 7.0), supplemented with 20 mM KCl. Spectra were obtained by subtracting the spectra recorded at 20 °C from those recorded at 90 °C. The presence of positive bands centered at ~243 and ~273 nm and a negative band around ~295 nm is indicative of G-quadruplex formation.

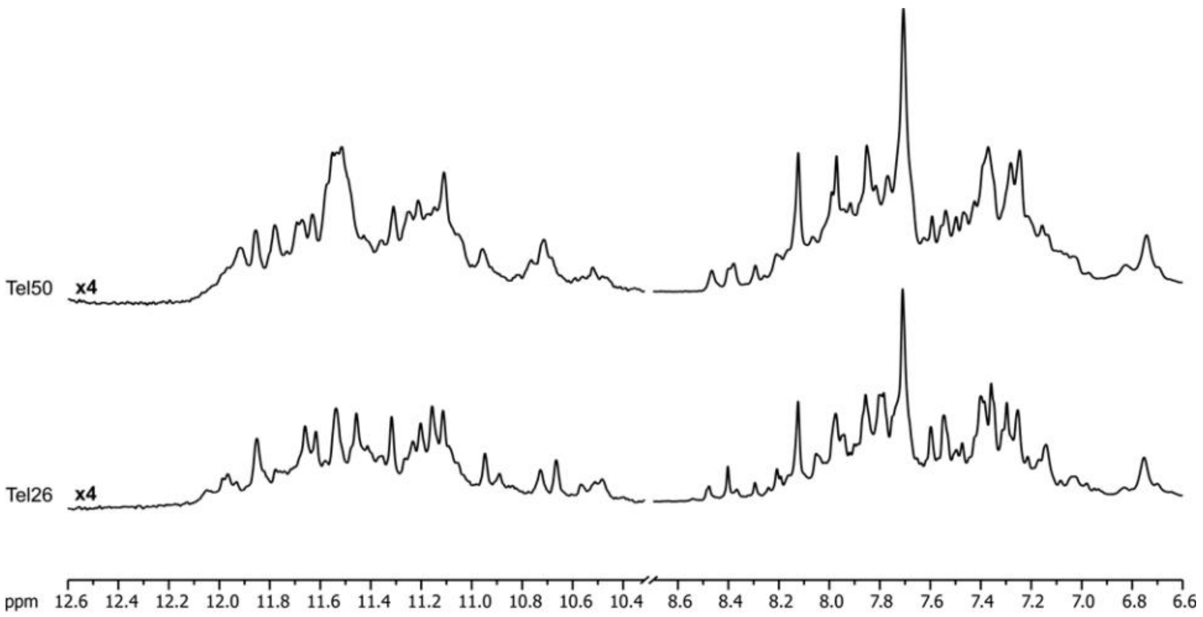

Figure S18. Imino and aromatic regions of the 1D ^{1}H NMR spectra of Tel26 and Tel50 in 5 mM KH_2PO_4/K_2HPO_4 buffer (pH 7.0), supplemented with 20 mM KCl. Spectra were recorded at 25 °C on a 600 MHz NMR spectrometer using an oligonucleotide concentration of 25 μM in 90% H_2O/10% D_2O.

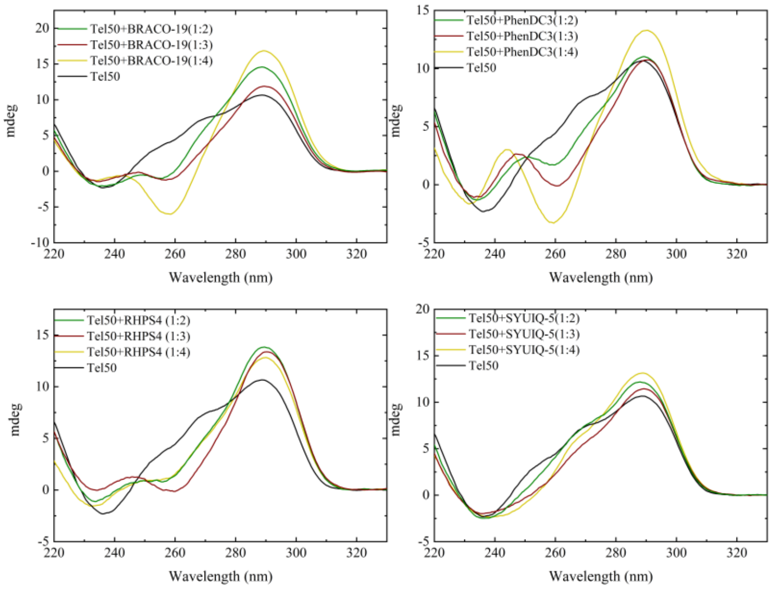

Figure S19. CD spectra of Tel50 in the absence and presence of the ligands at stoichiometric ratios of 1:2, 1:3, and 1:4. Tel50 + BRACO-19 (1:3) has a DNA concentration of 10 μM.

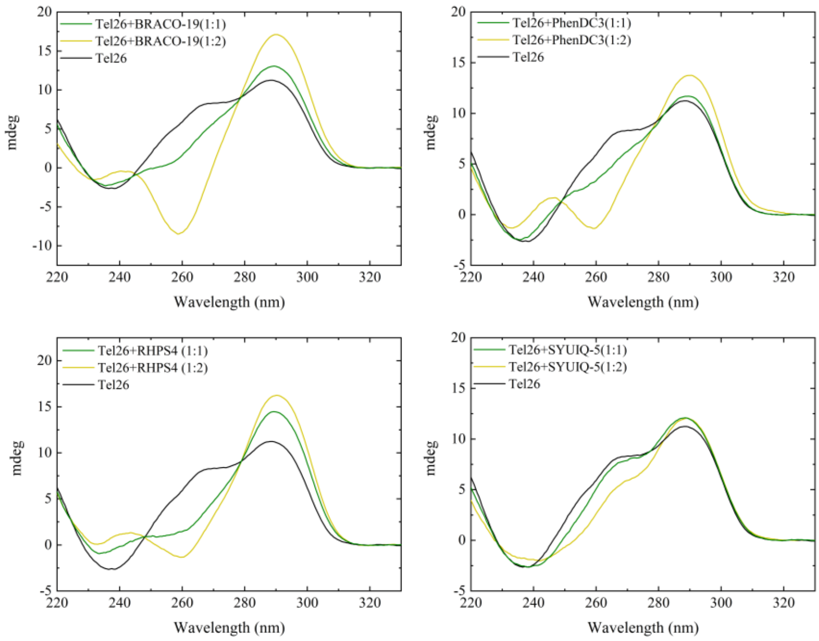

Figure S20. CD spectra of Tel26 in the absence and presence of the ligands at stoichiometric ratios of 1:1 and 1:2.

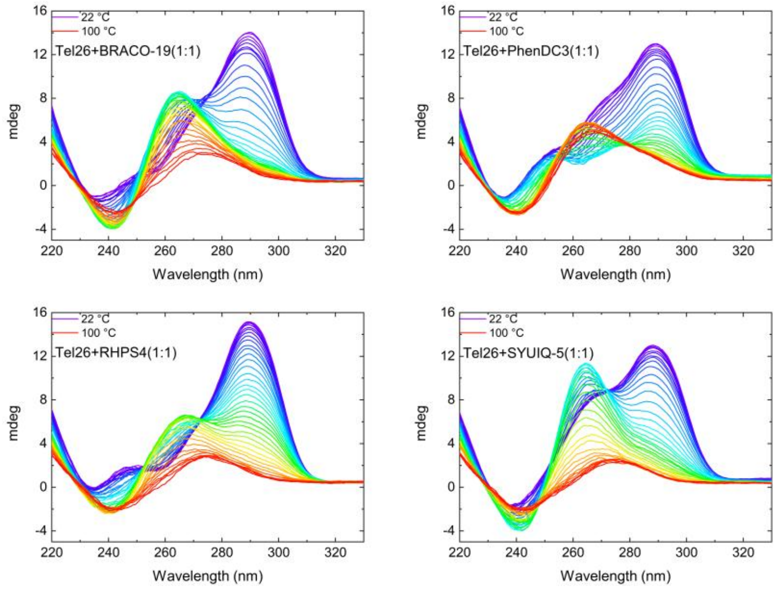

Figure S21. Temperature dependent unfolding of Tel26 in the presence of ligands assessed by CD spectroscopy. DNA-to-ligand stoichiometric ratios 1:1.

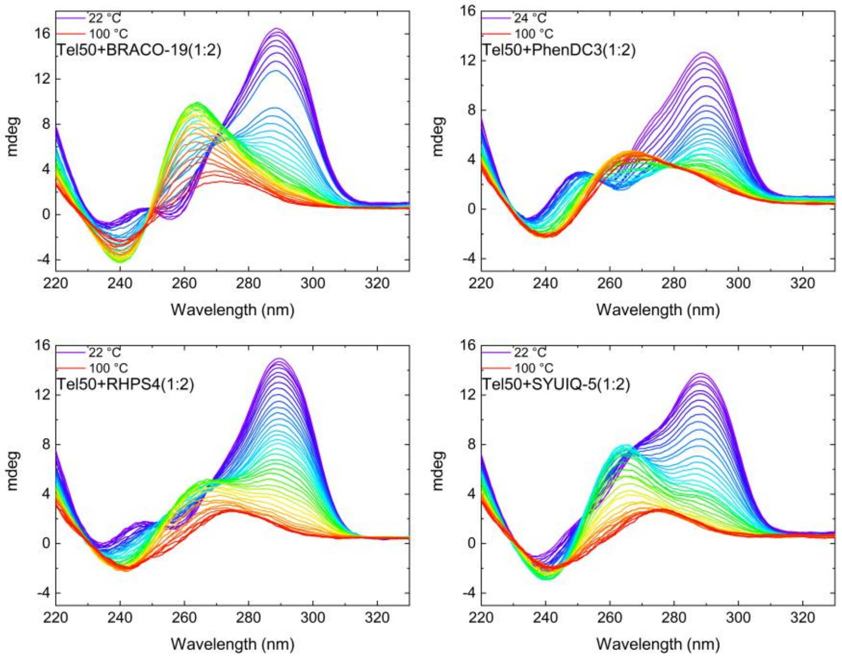

Figure S22. Temperature dependent unfolding of Tel50 in the presence of ligands assessed by CD spectroscopy. DNA-to-ligand stoichiometric ratios 1:2.

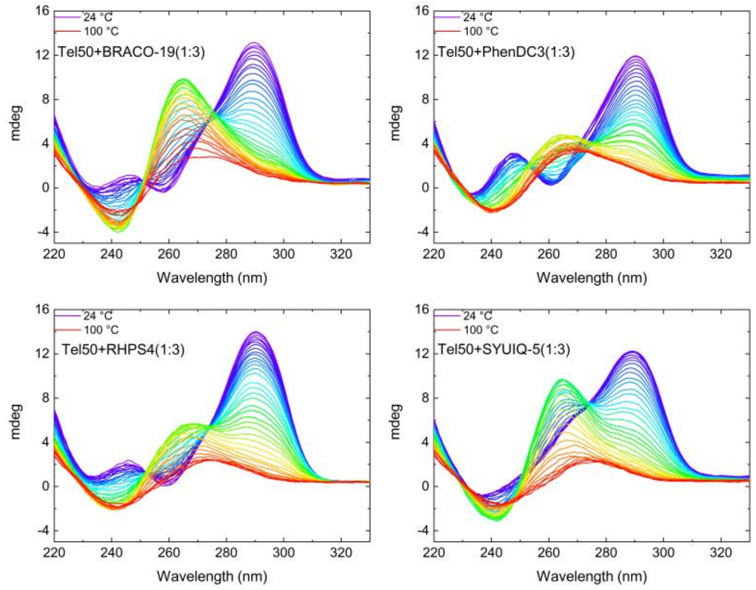

Figure S23. Temperature dependent unfolding of Tel50 in the presence of ligands assessed by CD spectroscopy. DNA-to-ligand stoichiometric ratios is 1:3.

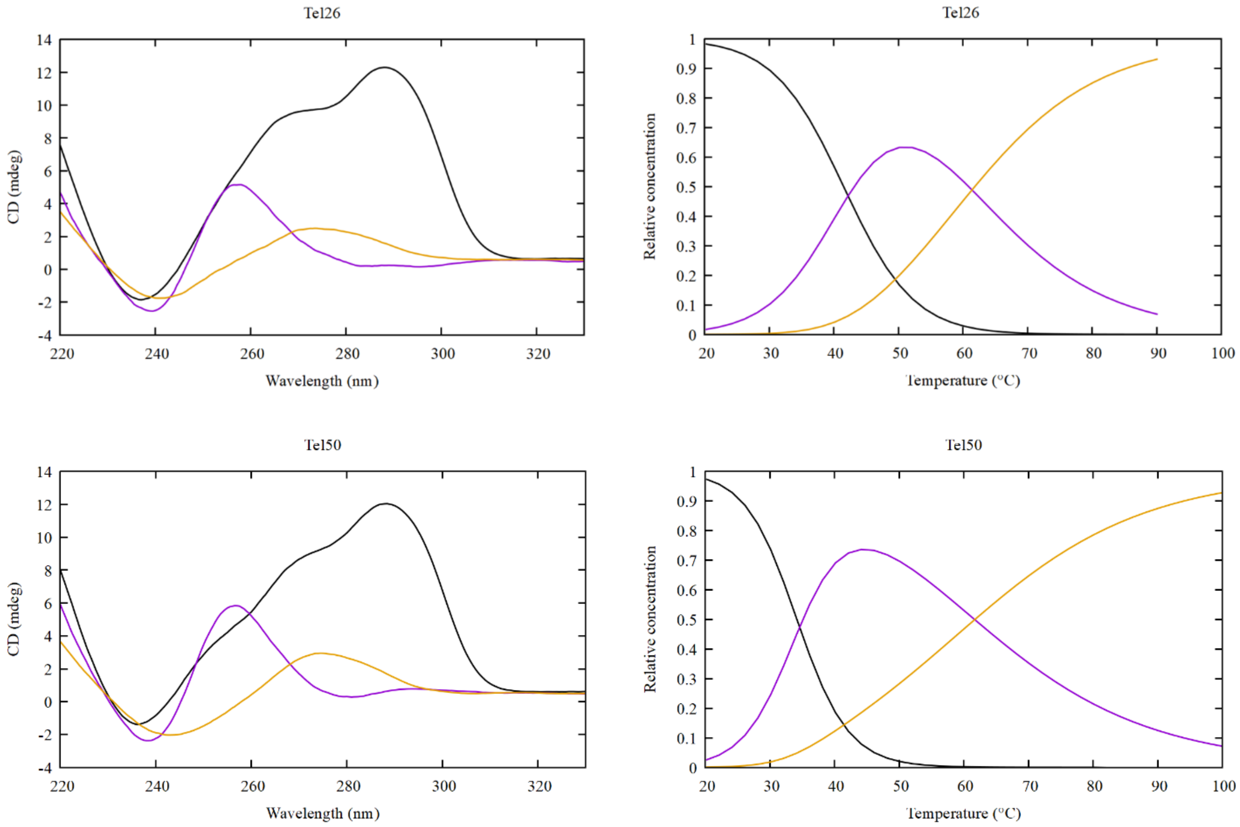

Figure S24. Results of the SVD analysis on CD data of Tel26 and Tel50. Left panels: spectra of significant species. Right panels: Relative concentration of significant species as a function of temperature. The folded state is shown in black, intermediate 1 in purple, intermediate 2 in light blue, and the unfolded state in yellow.

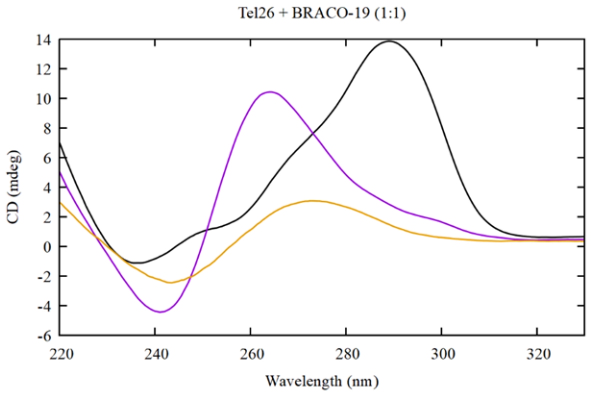
Tel26 + BRACO-19 (1:1)
CD (mdeg)
Wavelength (nm)

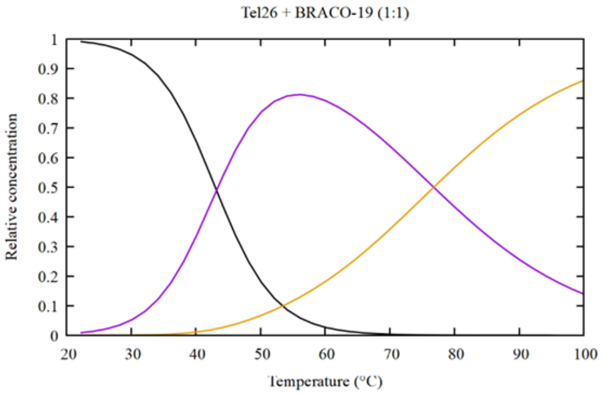
Tel26 + BRACO-19 (1:1)
Relative concentration
Temperature (°C)

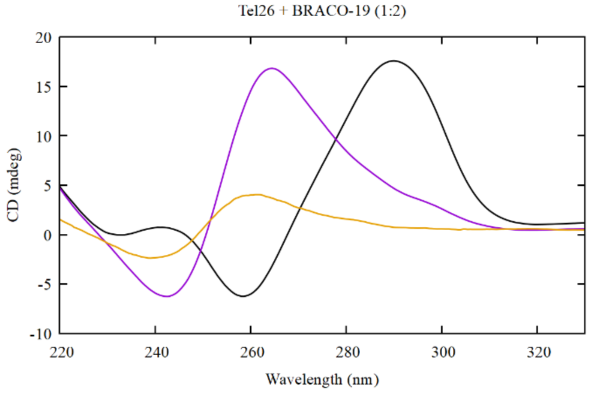
Tel26 + BRACO-19 (1:2)
CD (mdeg)
Wavelength (nm)

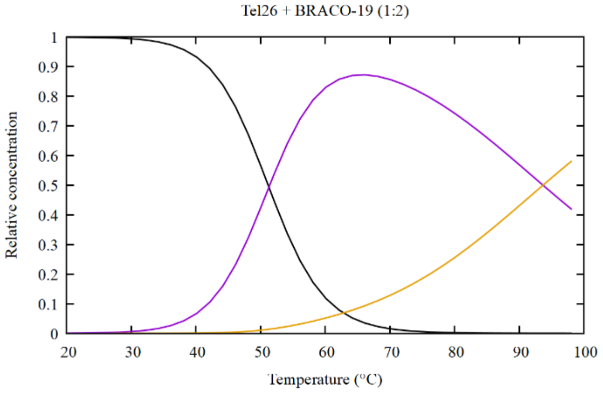
Tel26 + BRACO-19 (1:2)
Relative concentration
Temperature (°C)

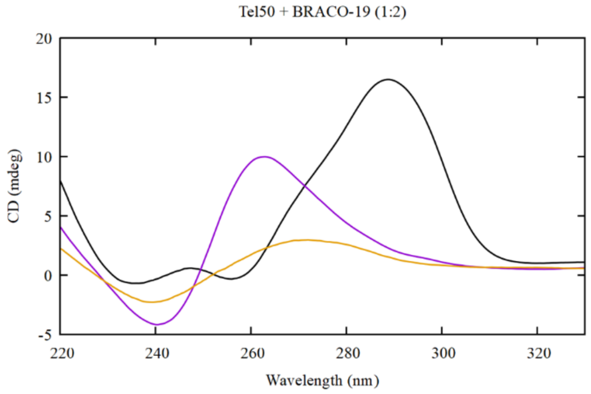
Tel50 + BRACO-19 (1:2)
CD (mdeg)
Wavelength (nm)

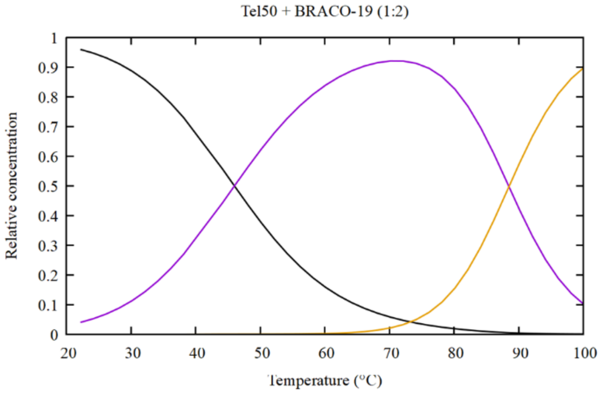
Tel50 + BRACO-19 (1:2)
Relative concentration
Temperature (°C)

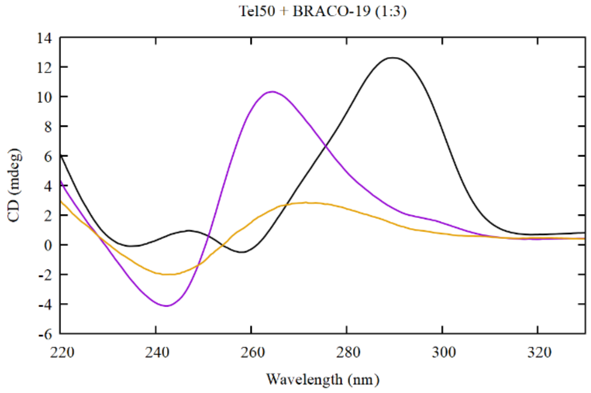
Tel50 + BRACO-19 (1:3)
CD (mdeg)
Wavelength (nm)

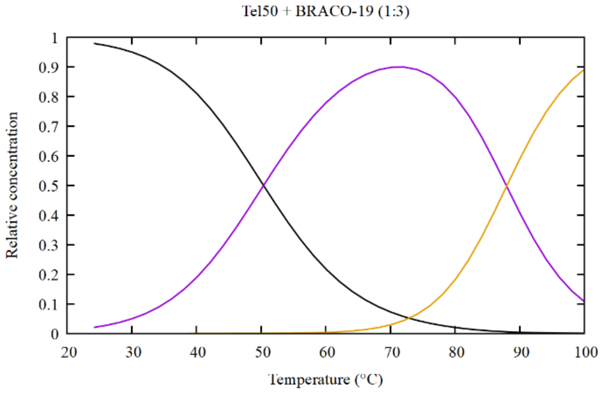
Tel50 + BRACO-19 (1:3)
Relative concentration
Temperature (°C)

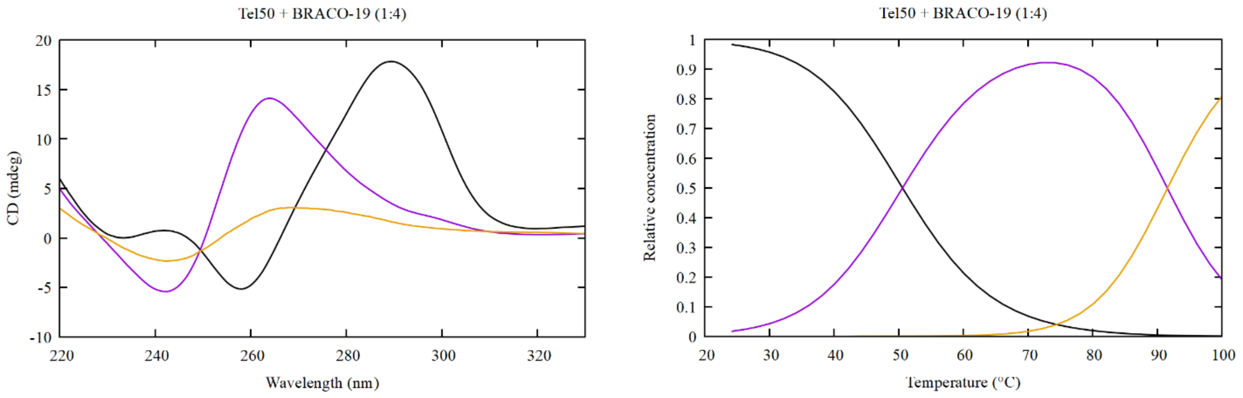

Figure S25. Results of the SVD analysis on CD data of Tel26+BRACO-19 and Tel50+BRACO-19. Left panels: Spectra of significant species. Right panels: Relative concentration of significant species as a function of temperature. The folded state is shown in black, intermediate 1 in purple, intermediate 2 in light blue, and the unfolded state in yellow.

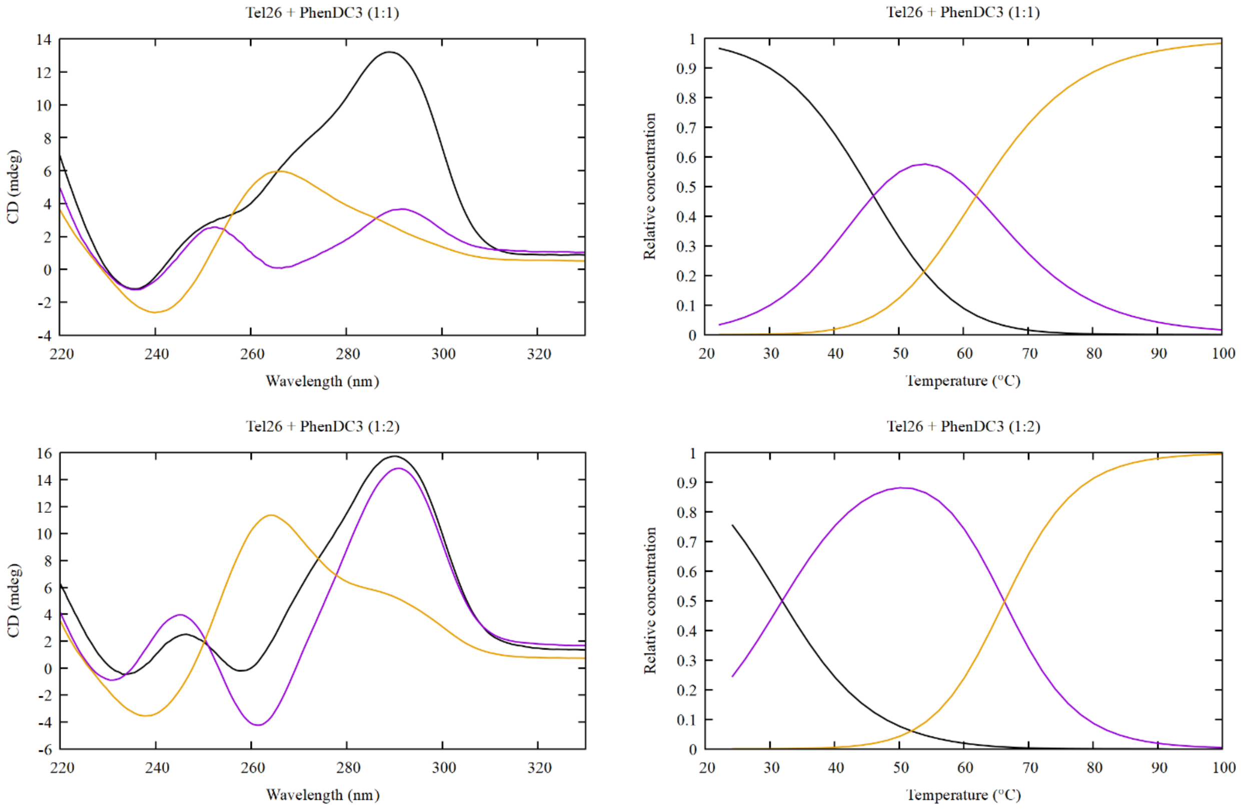

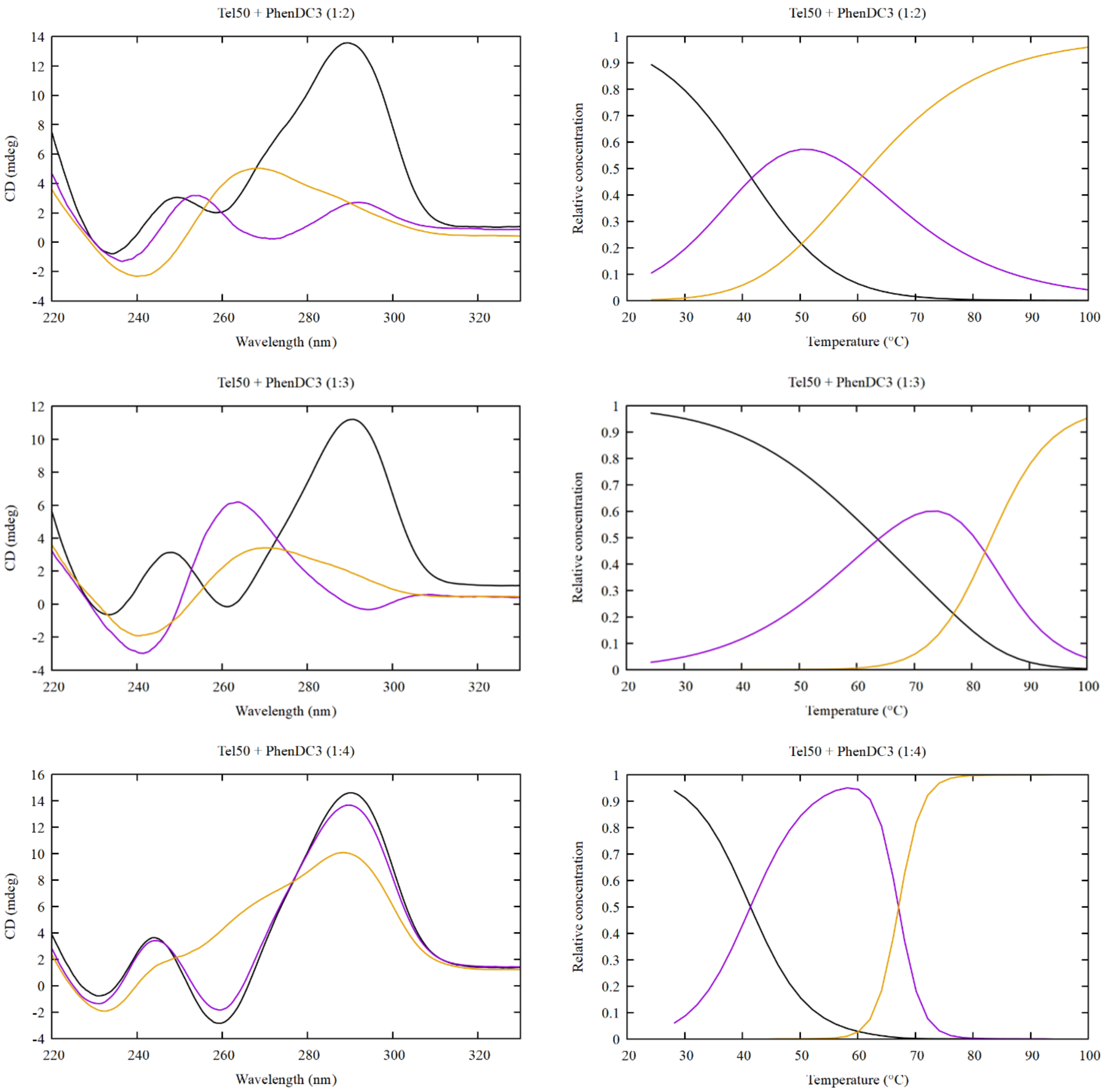

Figure S26. Results of the SVD analysis on CD data of Tel26+PhenDC3 and Tel50+PhenDC3. Left panels: Spectra of significant species. Right panels: Relative concentration of significant species as a function of temperature. The folded state is shown in black, intermediate 1 in purple, intermediate 2 in light blue, and the unfolded state in yellow.

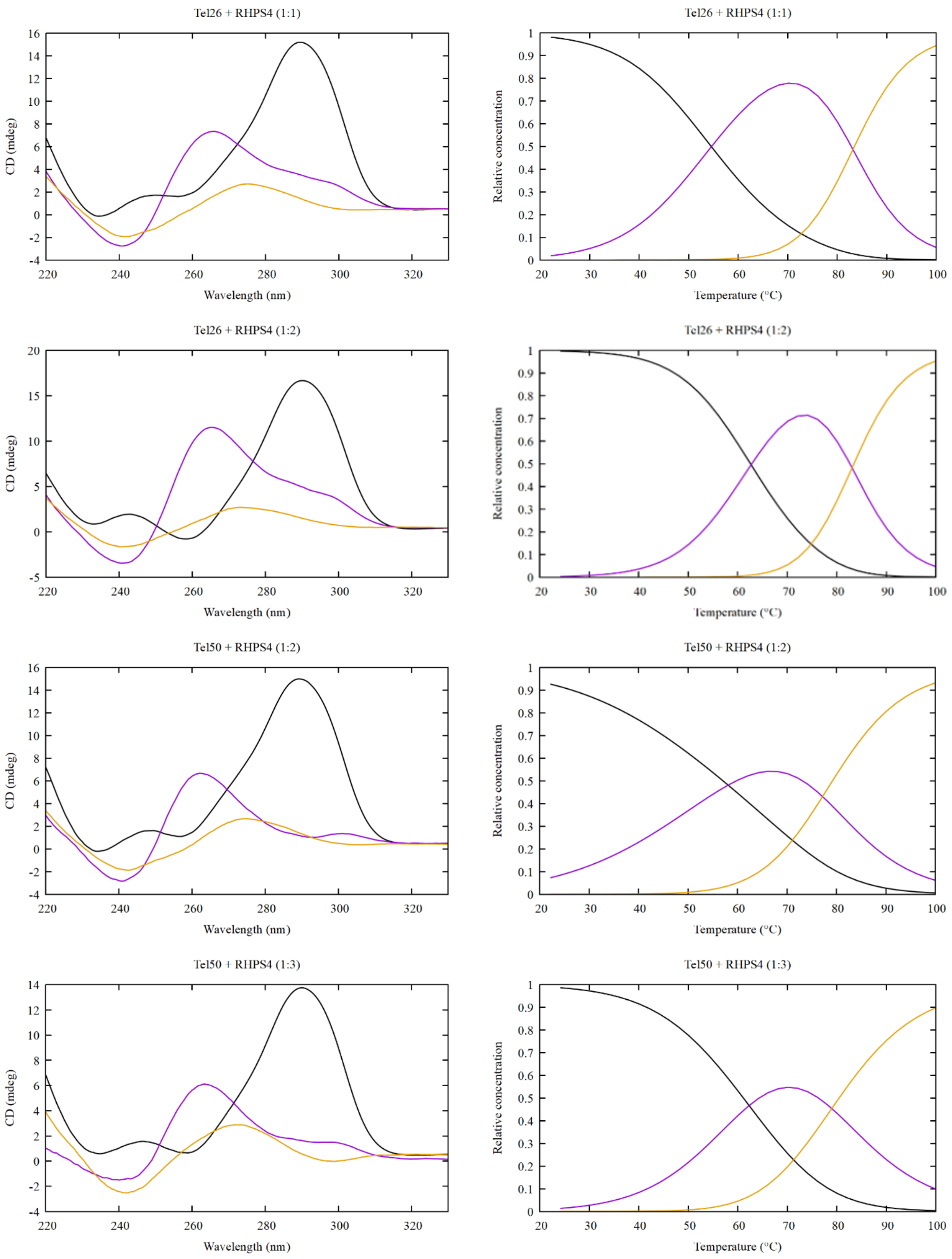

Tel26 + RHPS4 (1:1)
CD (mdeg)
Wavelength (nm)
Tel26 + RHPS4 (1:1)
Relative concentration
Temperature (°C)
Tel26 + RHPS4 (1:2)
CD (mdeg)
Wavelength (nm)
Tel26 + RHPS4 (1:2)
Relative concentration
Temperature (°C)
Tel50 + RHPS4 (1:2)
CD (mdeg)
Wavelength (nm)
Tel50 + RHPS4 (1:2)
Relative concentration
Temperature (°C)
Tel50 + RHPS4 (1:3)
CD (mdeg)
Wavelength (nm)
Tel50 + RHPS4 (1:3)
Relative concentration
Temperature (°C)

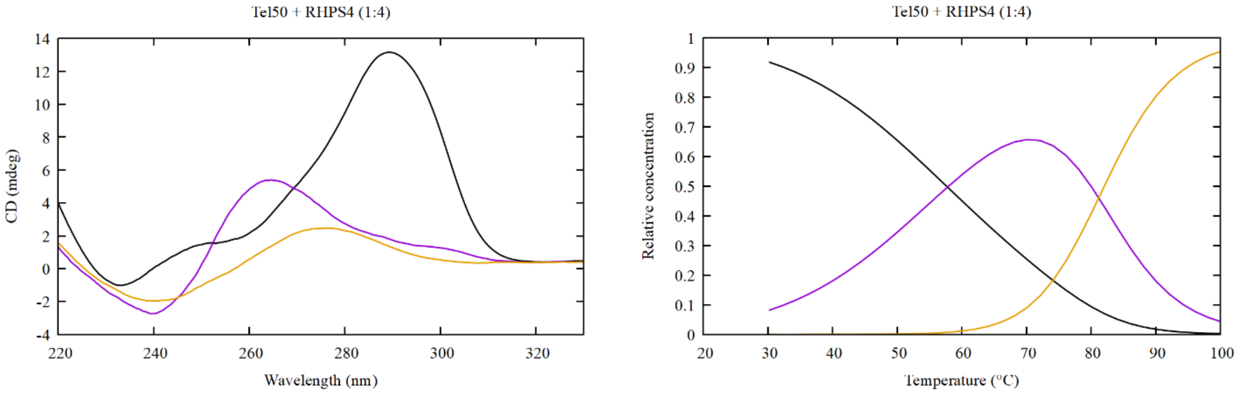

Figure S27. Results of the SVD analysis on CD data of Tel26+RHPS4 and Tel50+RHPS4. Left panels: Spectra of significant species. Right panels: Relative concentration of significant species as a function of temperature. The folded state is shown in black, intermediate 1 in purple, intermediate 2 in light blue, and the unfolded state in yellow.

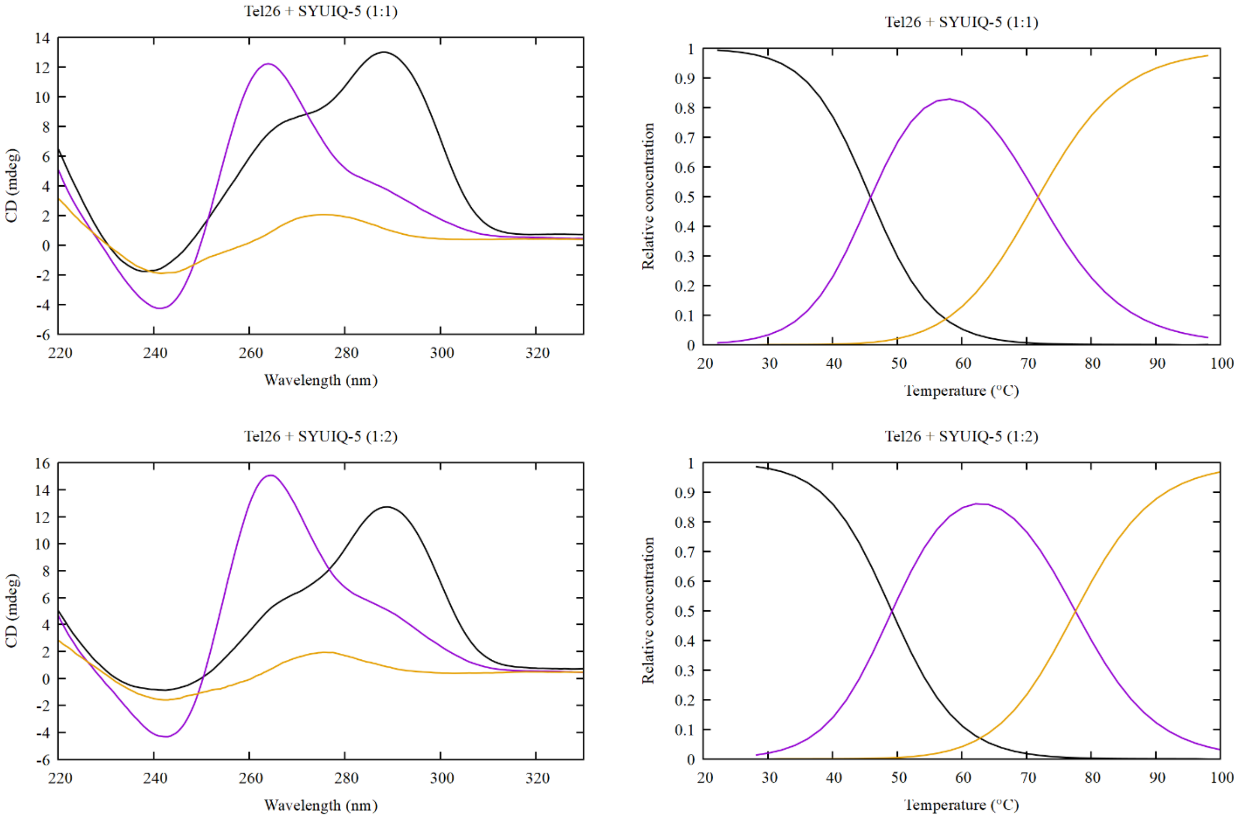

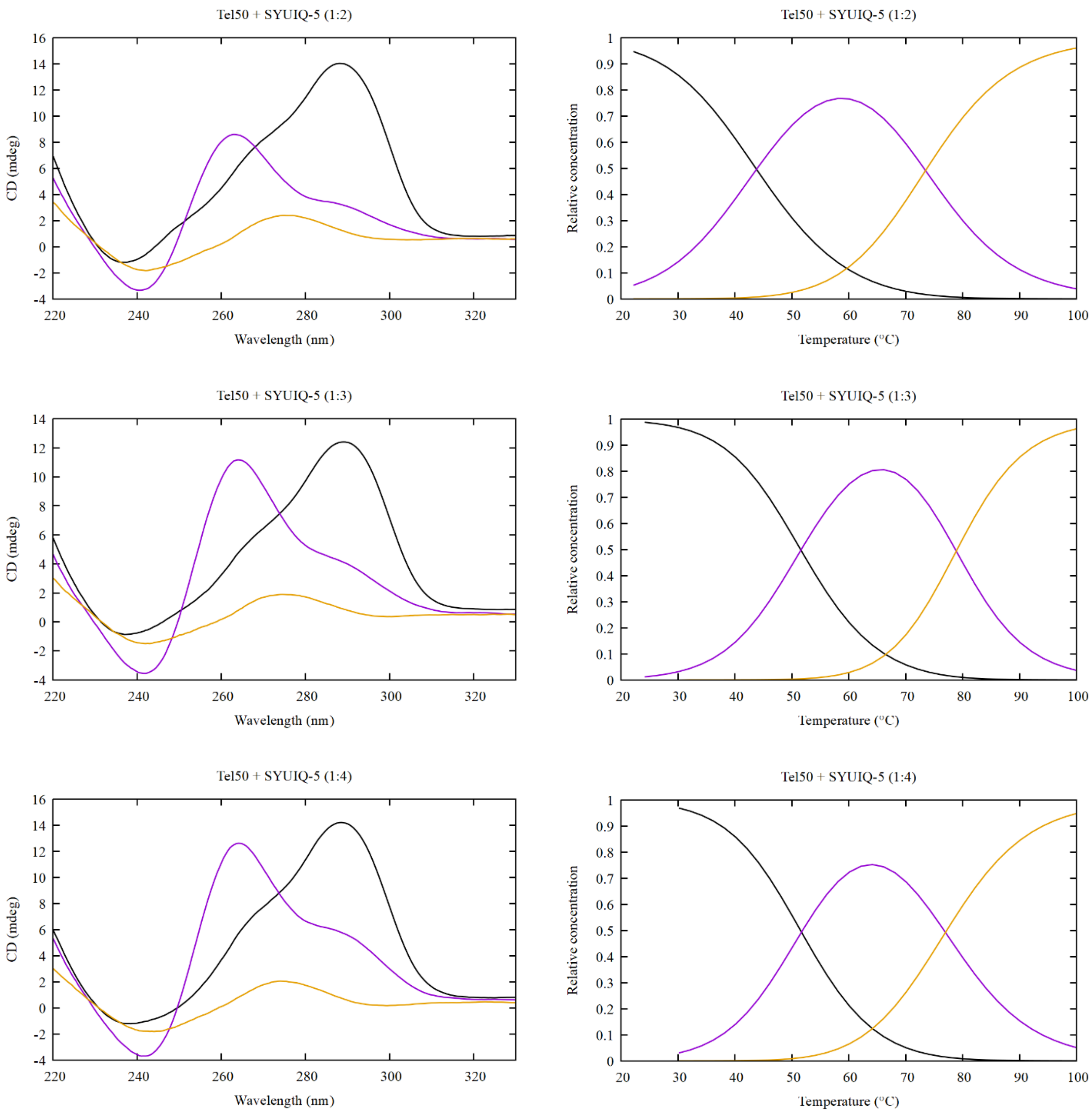

Figure S28. Results of the SVD analysis on CD data of Tel26+SYUIQ-5 and Tel50+SYUIQ-5. Left panels: Spectra of significant species. Right panels: Relative concentration of significant species as a function of temperature. The folded state is shown in black, intermediate 1 in purple, and the unfolded state in yellow.

TABLES

Table S1. R_g values of Tel26 and Tel26 + ligand complexes obtained from Guinier fits of the SAXS intensity profiles of the investigated samples are reported. The corresponding real-space R_g values derived from the *p(r)* distributions are also provided for comparison. The discrepancy between reciprocal space and real space R_g lies between 1% and 2%.

	Reciprocal space	Real space		
Sample	Guinier R_g (nm)	P(r) R_g (nm)	Dmax (nm)	χ^2/ndf
Tel26	1.31 ± 0.03	1.35 ± 0.01	4.8	1.008/945
Tel26+BRACO-19 1:1	1.31 ± 0.02	1.33 ± 0.01	4.7	0.971/945
Tel26+BRACO-19 1:2	1.31 ± 0.03	1.33 ± 0.01	4.8	1.062/945
Tel26+PhenDC3 1:1	1.32 ± 0.04	1.33 ± 0.01	4.7	1.013/945
Tel26+PhenDC3 1:2	1.31 ± 0.06	1.33 ± 0.01	4.7	1.020/945
Tel26+RHPS4 1:1	1.31 ± 0.03	1.33 ± 0.01	4.8	1.041/945
Tel26+RHPS4 1:2	1.30 ± 0.03	1.32 ± 0.01	4.7	0.957/945
Tel26+SYUIQ-5 1:1	1.31 ± 0.01	1.34 ± 0.01	4.7	0.977/945
Tel26+SYUIQ-5 1:2	1.32 ± 0.01	1.34 ± 0.01	4.7	1.024/945

Table S2. R_g values of Tel50 and Tel50 + ligand complexes obtained from Guinier fits of the SAXS intensity profiles of the investigated samples are reported. The corresponding real-space R_g values derived from the P(r) distributions are also provided for comparison. The discrepancy between reciprocal space and real space R_g lies between 3% and 5%, depending on the samples.

	Reciprocal space	Real space		
Sample	Guinier R_g (nm)	P(r) R_g (nm)	Dmax (nm)	χ^2/ndf
Tel50	2.10 ± 0.02	2.19 ± 0.01	7.3	1.035/736
Tel50+BRACO-19 1:2	2.02 ± 0.02	2.11 ± 0.01	6.8	1.025/736
Tel50+BRACO-19 1:3	2.00 ± 0.03	2.09 ± 0.01	6.7	1.058/736
Tel50+BRACO-19 1:4	1.98 ± 0.02	2.06 ± 0.01	6.7	1.083/736
Tel50+PhenDC3 1:2	2.03 ± 0.01	2.12 ± 0.01	6.7	1.217/736
Tel50+PhenDC3 1:3	1.99 ± 0.03	2.09 ± 0.01	6.7	1.159/726
Tel50+PhenDC3 1:4	1.97 ± 0.02	2.05 ± 0.01	6.5	1.071/722
Tel50+RHPS4 1:2	2.03± 0.01	2.12 ± 0.01	6.7	1.023/736
Tel50+RHPS4 1:3	2.01 ± 0.02	2.09 ± 0.01	6.7	1.018/736
Tel50+RHPS4 1:4	1.96 ± 0.01	2.05 ± 0.01	6.4	1.115/716
Tel50+SYUIQ-5 1:2	1.97 ± 0.03	2.05 ± 0.01	6.7	1.014/736
Tel50+SYUIQ-5 1:3	1.93 ± 0.03	1.99 ± 0.01	6.4	0.952/736
Tel50+SYUIQ-5 1:4	1.91 ± 0.01	1.97 ± 0.01	6.4	1.045/736

Table S3. Transition temperatures obtained with SVD analysis of Tel26 complexed with ligands at DNA:ligand stoichiometric ratio of 1:1 and 1:2. The melting temperatures are highlighted in red. n.d., not determined; the sample shows high stability and does not reach the melting transition.

Sample	T1(°C) (±1)	T2 (°C) (±1)	T3 (°C) (±1)
Tel26	42	62	
Tel26+BRACO-19 1:1	43	77	
Tel26+BRACO-19 1:2	51	94	
Tel26+PhenDC3 1:1	46	62	
Tel26+PhenDC3 1:2	32	66	n.d.
Tel26+RHPS4 1:1	54	83	
Tel26+RHPS4 1:2	63	83	
Tel26+SYUIQ-5 1:1	46	72	
Tel26+SYUIQ-5 1:2	49	78	

Table S4. Transition temperatures obtained with SVD analysis of Tel50 complexed with ligands at DNA:ligand stoichiometric ratio of 1:2, 1:3 and 1:4. The melting temperatures are highlighted in red. n.d., not determined; the sample shows high stability and does not reach the melting transition.

Sample	T1 (°C) (±1)	T2 (°C) (±1)	T3 (°C) (±1)
Tel50	34	62	
Tel50+BRACO-19 1:2	46	88	
Tel50+BRACO-19 1:3	50	88	
Tel50+BRACO-19 1:4	50	92	
Tel50+PhenDC3 1:2	41	61	
Tel50+PhenDC3 1:3	64	82	
Tel50+PhenDC3 1:4	41	67	n.d.
Tel50+RHPS4 1:2	58	77	
Tel50+RHPS4 1:3	62	79	
Tel50+RHPS4 1:4	58	81	
Tel50+SYUIQ-5 1:2	44	73	
Tel50+SYUIQ-5 1:3	51	79	
Tel50+SYUIQ-5 1:4	52	77	

Table S5. The root of the sum of squared deviations of the dichroism data [(Tel50 + ligand) − Tel50] and [(Tel26 + ligand) − Tel26].

Sample Tel50	RSQ (mDeg)	Sample Tel26	RSQ (mDeg)
Tel50+BRACO-19 1:4	64.931	Tel26+BRACO19 1:2	83.004
Tel50+PhenDC3 1:4	50.191	Tel26+PhenDC3 1:2	50.451
Tel50+RHPS4 1:4	26.475	Tel26+RHPS4 1:2	51.977
Tel50+SYUIQ-5 1:4	22.054	Tel26+SYUIQ 1:2	23.823
